# An Abrupt Aging of Dissolved Organic Carbon in Large Arctic Rivers

**DOI:** 10.1029/2020GL088823

**Published:** 2020-12-08

**Authors:** Melissa S. Schwab, Robert G. Hilton, Peter A. Raymond, Negar Haghipour, Edwin Amos, Suzanne E. Tank, Robert M. Holmes, Edward T. Tipper, Timothy I. Eglinton

**Affiliations:** ^1^ Department of Earth Sciences ETH Zurich Zurich Switzerland; ^2^ Department of Geography Durham University Durham UK; ^3^ Yale School of Forestry and Environmental Studies Yale University New Haven CT USA; ^4^ Laboratory of Ion Beam Physics ETH Zurich Zurich Switzerland; ^5^ Aurora Research Institute Inuvik Northwest Territories Canada; ^6^ Department of Biological Sciences University of Alberta Edmonton Alberta Canada; ^7^ Woods Hole Research Center Falmouth MA USA; ^8^ Department of Earth Sciences University of Cambridge Cambridge UK

**Keywords:** dissolved organic carbon, Mackenzie River, permafrost, discharge, warming

## Abstract

Permafrost thaw in Arctic watersheds threatens to mobilize hitherto sequestered carbon. We examine the radiocarbon activity (F^14^C) of dissolved organic carbon (DOC) in the northern Mackenzie River basin. From 2003–2017, DOC‐F^14^C signatures (1.00 ± 0.04; *n* = 39) tracked atmospheric ^14^CO_2_, indicating export of “modern” carbon. This trend was interrupted in June 2018 by the widespread release of aged DOC (0.85 ± 0.16, *n* = 28) measured across three separate catchment areas. Increased nitrate concentrations in June 2018 lead us to attribute this pulse of ^14^C‐depleted DOC to mobilization of previously frozen soil organic matter. We propose export through lateral perennial thaw zones that occurred at the base of the active layer weakened by preceding warm summer and winter seasons. Although we are not yet able to ascertain the broader significance of this “anomalous” mobilization event, it highlights the potential for rapid and large‐scale release of aged carbon from permafrost.

## Key Points

- A widespread pulse of aged dissolved organic carbon (DOC) occurred in the Mackenzie River and its tributaries in June 2018
- Export of aged DOC is consistent with a prolonged warming period and the formation of supra‐permafrost taliks
- Mobilization of aged DOC and nitrate suggests percolation of supra‐permafrost groundwater through previously frozen soil layers

## 1 Introduction

The northern circumpolar permafrost regions contain 44% of the global soil organic carbon stock within its top 3 m, corresponding to approximately twice the amount of carbon present in the atmosphere (Hugelius et al., 2014; Schuur et al., 2008). Organic‐rich permafrost soils have accumulated over millennia in peat deposits and deltaic sediments (Zimov et al., 2006) with subzero temperatures acting to inhibit microbial decomposition and preserve organic matter (Ping et al., 2011). Warming can lead to thawing and destabilization of these perennially frozen soils (Jorgenson et al., 2006), rendering this vast, aged carbon stock vulnerable to change (Schuur et al., 2015). Permafrost thaw exposes any associated organic matter to mechanical and thermal erosion and can promote microbial decomposition and/or photodegradation (Ward & Cory, 2016) that subsequently leads to the release of CO_2_ or methane (Schuur et al., 2008). In the last 50 years, mean annual air temperatures in northern Canada have increased by 2.3°C (and winter temperatures by 4.3°C), roughly 2–3 times the global average (Bush & Lemmen, 2019). Estimates of the impact of permafrost degradation on high‐latitude soil carbon stocks predict a loss of between ~10 and ~170 PgC by the end of this century (Koven et al., 2015; Schaefer et al., 2014; Schuur et al., 2015), potentially adding to atmospheric greenhouse gas burdens and exacerbating future climate change. Given these projections, tracking the fate of this aged carbon pool, and constraining its response to both gradual and abrupt warming and permafrost thaw, remains a priority (Turetsky et al., 2019).

Along with direct release to the atmosphere, aged carbon mobilized during permafrost thaw can enter surface waters (Plaza et al., 2019) in the form of dissolved organic carbon (DOC) (operationally defined as <0.2 to <0.7 μm), while particulate organic carbon (POC) is largely supplied to freshwater systems by thermokarst and bank erosion (Jorgenson et al., 2006; Vonk et al., 2015). The degradation of permafrost can increase the thickness of the active layer and result in the loss of ground ice, leading to the formation of thaw slumps, taliks, and thermokarst wetlands (Vonk et al., 2015). Deeper groundwater flow paths contribute to the development of supra‐permafrost taliks (areas of unfrozen ground) and connect laterally to the drainage network (Woo, 2012). In early spring, the infiltration of meltwater into permafrost soils can activate hydrologically connected lateral perennial thaw zones (Connon et al., 2018; Walvoord et al., 2019). The flow through supra‐permafrost taliks may mobilize DOC from previously frozen soils, well before seasonal permafrost begins to thaw (Walvoord et al., 2019). The sources and flux of DOC in Arctic rivers, which has garnered interest due to its potential reactivity (Cory et al., 2013; Holmes et al., 2008; Mann et al., 2015; Vonk et al., 2013) and its intrinsic link to hydrological pathways, may be modified by such ground thaw (Fouché et al., 2020; Spencer et al., 2015). As water flow accesses deeper layers, a decrease in ^14^C activity of DOC has been projected (Frey & McClelland, 2009; Schuur et al., 2015; Tank et al., 2012). Old DOC has previously been identified at small scales, for example, in permafrost seeps and small streams (Dean et al., 2018; Mann et al., 2015; Neff et al., 2006; Vonk et al., 2013). However, in the large Arctic rivers (Yenisey, Lena, Ob', Mackenzie, Yukon, Kolyma) the ^14^C activity of DOC has been shown to contain “bomb” carbon stemming from atmospheric nuclear weapons testing in the middle‐twentieth Century, implying that young and rapidly cycling organic matter—likely derived from surface vegetation and soils—dominates DOC in large rivers (Raymond et al., 2007; Spencer et al., 2015).

In addition to the mobilization of aged carbon pools, permafrost thaw can result in enhanced weathering of mineral soils and bedrock, further modifying riverine aquatic chemistry (e.g., Frey & McClelland, 2009; Kokelj & Burn, 2005; O'Donnell et al., 2016; Tank et al., 2016; Vonk et al., 2015). As such, the export of major ions (e.g., Ca^2 +^ and SO_4_^2 −^ ) which can be enriched in deeper mineral soils is expected to increase in all circumpolar rivers as permafrost degradation persists (Frey & McClelland, 2009; Tank et al., 2012, 2016). In contrast, dissolved nitrogen in the form of nitrate, NO_3_^−^, is bioactive and influenced by cycling through vegetation and microbial respiration. Elevated riverine NO_3_^−^ concentrations have been linked to thaw slumps and thermokarst gullies (Bowden et al., 2008; Harms et al., 2014), as well as enhanced microbial nitrification and subsequent leaching (Fouché et al., 2017; Louiseize et al., 2014). As such, these dissolved inorganic ions can provide additional insight on sources and pathways of the products of permafrost thaw.

**Figure 1:**
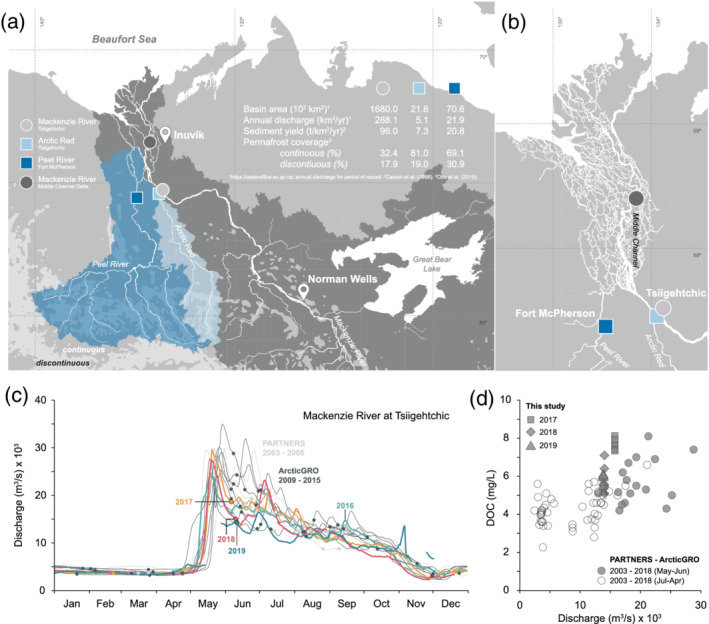
(a) River depth profile sampling locations along the Mackenzie River (circle) in the Delta (dark gray) and at Tsiigehtchic (light gray) complemented by the major tributaries, the Arctic Red (light blue square) and the Peel River (dark blue diamond). (b) Mackenzie Delta closeup. (c) Discharge at the Mackenzie River at Tsiigehtchic from 2003 to 2019 (http://www.wateroffice.ec.gc.ca). Dots denote sampling dates. (d) DOC concentrations as function of discharge. Shapes denote different sampling periods.

The Mackenzie River (1.8 × 10^6^ km^2^) is a major carbon source to the Arctic Ocean (Hilton et al., 2015) and has experienced increasing DOC fluxes over the last four decades (Tank et al., 2016). About 50% of the basin lies within continuous and discontinuous permafrost zones (Obu et al., 2019), with its two large, northern tributaries (Arctic Red: 21.8 × 10^3^ km^2^; Peel River: 70.6 × 10^3^ km^2^) draining almost exclusively continuous permafrost (Figure 1a). The northern part of the basin has been particularly affected by climate change over the instrumental record and has been identified as a region whose carbon stocks are under threat from permafrost thaw (Bush & Lemmen, 2019; Kokelj et al., 2017). Here, we explore the hypothesis that enhanced regional warming could modify transport pathways and the contribution of carbon stored in permafrost soils. We investigate the ^14^C age of DOC and the flux of major ions carried by the Mackenzie River and its northern tributaries to identify potential processes impacting the export of DOC in a high‐latitude river.

## 2 Methods

We collected river water in May–June 2017–2019, shortly after ice breakup at the high/receding water stage (Figure 1c), for the Mackenzie River at Tsiigehtchic, in the Delta, and for the Peel and Arctic Red Rivers (Figure 1a). In order to assess vertical variation, we used a modified horizontally mounted ~5.1 L Niskin bottle to recover water from different depths (Hilton et al., 2015). River water was transferred to sterilized plastic bags, weighed, stored in the dark, and filtered within 48 hr using precleaned filtration units (2017–2018) or Teflon‐lined steel units (2019) through polysethersulfone filters (PES; Ø 142 mm, 0.22 μm). Filtered water was collected for DOC in 120 ml pre‐combusted (450°C, 6 hr) amber bottles, acidified to pH ~2 with 85% H_3_PO_4_ (120 μl), and stored cooled and in the dark. Aliquots for ion analysis were collected in acid‐washed high density polyethylene (HDPE) bottles. Sediment‐laden filters were folded, carefully placed in pre‐combusted aluminum foil envelops and immediately frozen.

Major ions were measured at Durham University and ETH Zurich using a Dionex Ion Chromatography system (DX‐120, Thermoscientific) with an analytical reproducibility of 5%. In order to help track water pathways, we utilize ratios of [Ca^2 +^ ]/[Cl^−^] and [NO_3_^−^]/[Ca^2 +^ ]. Cl^−^ is considered as relatively conservative ion in aquatic environments, with minor engagement in biological and geochemical processes and sourced mainly from rainwater (Gaillardet et al., 1999). Dissolved Ca^2 +^ is mostly derived from chemical weathering of carbonate minerals in the Mackenzie River basin (Gaillardet et al., 1999; Millot et al., 2003; Tank et al., 2012). As such, [Ca^2 +^]/[Cl^−^] ratios can be used to compare the relative mobilization of chemical weathering products between sampling periods. In contrast, NO_3_^−^ is strongly linked to nutrient cycling in organic matter in near‐surface soils. Microbial mineralization of organic matter accumulates NO_3_^−^ during the winter months when the uptake by plants is diminished (Edwards & Jefferies, 2013; Treat et al., 2016). The [NO_3_^−^]/[Ca^2 +^] ratio therefore allows us to assess the relative influence of organic matter cycling versus mineral weathering, albeit with the recognition that NO_3_^−^ is not necessarily conservative and release from the landscape may not cascade to increases in NO_3_^−^ in river channels (Harms & Jones, 2012; Wickland et al., 2018).

DOC concentration measurements were conducted using a Shimadzu system (TOC‐L Series) at the Department of Environmental System Science at ETH Zurich. DOC (10–53 μgC) was converted to CO_2_ using a wet chemical oxidation approach (Lang et al., 2012, 2016). Prior to the oxidation inorganic CO_2_ was removed by purging. Evolved CO_2_ from DOC oxidation was analyzed using a mini carbon dating accelerator mass spectrometer (MICADAS AMS) system equipped with a gas‐accepting ion source at the Laboratory for Ion Beam Physics (LIP) at ETH Zurich. Blank assessment was based on the repeated measurements of sucrose (Sigma, δ^13^C = −12.4‰ VPDB, F^14^C = 1.053 ± 0.003) and phthalic acid (Sigma, δ^13^C = −33.6‰ VPDB, F^14^C < 0.0025) standards. The evaluation of constant contamination described in Haghipour et al. (2019) amounted to ~1 μgC.

Supporting information provides details of sample collection and analyses of various carbon species (DOC and POC), nutrients, and major ions retrieved since 2003 near the apex of the Mackenzie Delta (at Tsiigehtchic, upstream of the Arctic Red and Peel inputs) by the Pan‐Arctic River Transport of Nutrients, Organic Matter, and Suspended Sediments (PARTNERS) and the Arctic Great Rivers Observatory (ArcticGRO; www.arcticgreatrivers.org) projects.

## 3 Results

The samples from the Mackenzie River at Tsiigehtchic collected in June 2017, 2018, and 2019 were taken at similar levels of water discharge and similar points on the hydrograph (i.e., shortly after highest peak) to those collected from 2003–2016, although 2018 and 2019 had the lowest June discharge in the data set (Figure 1c). For the Peel River, the ice breakup is typically a few days earlier in May, and the 2017–2019 samples were collected at similar times relative to the discharge peak (Figure S1).

The June 2017, 2018, and 2019 sampling campaigns resulted in similar DOC concentrations for the Mackenzie River at Tsiigehtchic (6.4 ± 0.9 mg/L, *n* = 18; Table S1) (± SD), which is within the variability of the average of June samples from 2003 to 2017 from the PARTNERS‐ArcticGRO campaign (5.7 ± 1.0 mg/L, *n* = 17) (Figure 1c). The DOC concentration for 2017–2019 samples was also similar for the Mackenzie River in the Delta (7.3 ± 0.5 mg/L, *n* = 22), the Peel River (3.9 ± 0.5 mg/L, *n* = 9), and the Arctic Red River (9.5 ± 2.0, *n* = 8).

**Figure 2:**
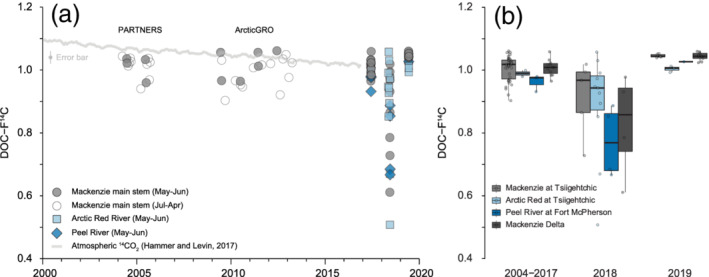
Radiocarbon activity, expressed as fraction modern (F^14^C), of DOC over time in the Mackenzie River: (a) F^14^C of DOC. Circles are samples from the Mackenzie River at Tsiigehtchic and the main channel in the Delta, with filled circles samples collected in May and June. Samples from the Arctic Red and Peel Rivers are also shown for 2017–2019. Data from 2004 to 2013 are provided by the ArcticGRO database. The atmospheric bomb ^14^C curve is shown as a light gray line (Hammer & Levin, 2017). The error bar in the top left corner represents the mean standard deviation of ^14^C measurements. (b) Boxplot of F^14^C for different years as a function of sampling location, with the median (black line), first and third quartiles (box), and confidence interval (lines) shown.

From 2003–2013 for the Mackenzie River at Tsiigehtchic, DOC‐F^14^C values in May and June ranged between 1.06 and 0.96, with an average DOC‐F^14^C value of 1.01 ± 0.04 (*n* = 10). In 2017, the F^14^C values were similar for both sites on the Mackenzie River (Figure 1a and Table S1) and the Arctic Red and Peel Rivers, with an average DOC‐F^14^C = 1.00 ± 0.03 (*n* = 22), and showed little variability with depth in the river (Figure S2). In June 2018, for the Mackenzie River at Tsiigehtchic, DOC‐F^14^C values ranged from 1.02 to 0.73. Aged DOC was present throughout the Mackenzie River system (Figure 2), with an average DOC‐F^14^C value across all sample of 0.85 ± 0.16 (*n* = 28; 1,306 ± 1,530 ^14^C years). In 2018, the DOC was even more ^14^C‐depleted (values as low as 0.51) in the Arctic Red and Peel Rivers. The Peel and Arctic Red basins drain higher latitudes, with higher proportions of continuous and discontinuous permafrost cover than the Mackenzie River at Tsiigehtchic (Figure 1a). These rivers join the main stem and combine in the Mackenzie Delta (Middle Channel), where old DOC was also prevalent in 2018. It is important to note that the Peel, Arctic Red, and Mackenzie Rivers at Tsiigehtchic drain different basins and collectively encompassing a large drainage area (Figure 1a).

In June 2019, DOC‐F^14^C values resembled those of years prior to 2017 (average DOC‐F^14^C = 0.99 ± 0.01, *n* = 20). Nonparametric Kruskal‐Wallis tests confirm the statistically significant difference between the ^14^C content of samples collected in 2018 from the Mackenzie River at Tsiigehtchic and the Mackenzie Delta compared to those sampled in 2003–2017 and in 2019 (Table S2).

**Figure 3:**
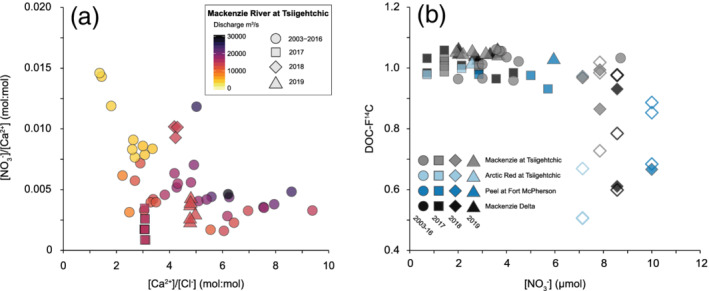
(a) Dissolved inorganic species in Mackenzie River at Tsiigehtchic. Relationship between [Ca^2 +^]/[Cl^−^] and [NO_3_^−^]/[Ca^2 +^]. Circles represent the values from 2003–2016 (ArcticGRO database), squares 2017, diamonds 2018, and triangles 2019. Symbols are color coded for discharge (m^3^/s). (b) Radiocarbon activity (expressed as F^14^C) of DOC versus NO_3_^−^ (μmol) during the spring freshet (May and June). The Mackenzie River samples at Tsiigehtchic and the Delta are indicated in gray and black, whereas the Arctic Red and Peel Rivers are depicted in light and dark blue, respectively. Open symbols represent NO_3_^−^ values integrated over depth.

Nitrate concentrations ([NO_3_^−^]) in the Mackenzie River at Tsiigehtchic (1.53 ± 0.6 μmol, *n* = 7) were lower during the spring freshet in 2017 than the long‐term observations of the PARTNERS‐ArcticGRO projects (3.9 ± 1.5 μmol, *n* = 17). Similar [NO_3_^−^] are observed in Arctic Red (1.8 ± 1 μmol, *n* = 3) and Mackenzie Delta (2.4 ± 1.1 μmol, *n* = 18) waters, while the Peel River supplied 4.9 ± 1.2 μmol (*n* = 5) to the Delta in 2017 and 2019. In 2018, we observe a twofold to threefold increase in the [NO_3_^−^] in the Mackenzie River and all subcatchments (Figure 3 and Table S3). During the spring freshet, we note a significant negative correlation between log‐transformed [NO_3_^−^] and DOC‐F^14^C (*r* = −0.54, *n* = 60, *p* < 0.001).

## 4 Discussion

Prior to this study, reported DOC‐F^14^C values in the large Arctic Rivers (Yenisey, Lena, Ob', Yukon, Kolyma) ranged from 0.82 to 1.16 (Barnes et al., 2018), comparable to those of the Mackenzie River up to 2017 (DOC‐F^14^C: 0.80–1.1; Figure 2) and similar to atmospheric ^14^CO_2_ (Dean et al., 2020; Raymond et al., 2007; Spencer et al., 2015). This suggests that the DOC pool derives from recently formed biomass, for example, plants, surficial soils, and aquatic productivity (Raymond & Bauer, 2001). Aged DOC has been detected in permafrost seeps and small headwater streams in the Arctic (Dean et al., 2018; Mann et al., 2015; Neff et al., 2006; Vonk et al., 2013). However, aged DOC has not previously been documented in larger Arctic rivers, implying efficient removal during riverine transfer (Holmes et al., 2008; Mann et al., 2015; Vonk et al., 2013) or dilution by modern DOC (Dean et al., 2018). Moderately aged DOC from large Arctic rivers has been attributed to the maximum expansion of the active layer and a deeper penetration of groundwater (Barnes et al., 2018). The presence of aged DOC has been observed in low latitude (Moore et al., 2013) and temperate watersheds (Evans et al., 2014) and linked to anthropogenic disturbance (Butman et al., 2015; Drake et al., 2019; Griffith et al., 2009). In the context of this prior work, we discuss mechanisms and their drivers that can explain two important features of the decadal time series from the Mackenzie River: (i) the ^14^C depletion of DOC in June 2018 across three separate catchment areas and (ii) the return to ^14^C‐enriched DOC in June 2019.

### 4.1 The Timing of Sampling

DOC mobilization and supply to rivers can vary as a function of precipitation (Raymond, 2005; Stubbins et al., 2012) and water discharge (Masiello & Druffel, 2001; Tittel et al., 2015). Broad‐scale changes in hydrology associated with a shift in DOC source and flux at the time of sampling might explain the June 2018 aged DOC signal. The Mackenzie River hydrograph is regulated by the onset of thaw and has a remarkably consistent annual pattern (Figure 1c). Snow and ice melt induce a rapid increase in streamflow in spring (freshet) which gradually subsides over subsequent months. During the spring freshet, the concentration, composition, and average age of DOC could vary with discharge, perhaps in response to a “pulse‐shunt”‐like behavior (Raymond et al., 2016). Snowmelt triggers the pulse‐like mobilization and transport of DOC and other nutrients to streams and rivers. Associated high‐water velocities shunt solutes rapidly downstream, reducing their residence time in the fluvial network and hence the potential for microbial or photochemical remineralization.

However, no relationship between water discharge and DOC‐F^14^C emerges in the 2003–2017 and 2019 data from the Mackenzie River at Tsiigehtchic. Instantaneous DOC fluxes measured in June 2018 are similar to those in June 2017 and 2019, with differences in flux mainly controlled by the water yield (Figures S3 and S4). DOC concentrations are similar, both as a function of water column depth and over the studied time period (Figure S5). It is therefore difficult to attribute the June 2018 ^14^C depletion of DOC to a feature of the typical annual hydrograph that has been missed in past sampling. When we consider the aged DOC input in the Peel and Arctic Red Rivers (which drain large, separate watersheds), it suggests that any change in the routing of DOC in June 2018 must be widespread. While we cannot constrain its duration, the sampling of these different rivers took place over 7 days. More frequent samples collected in May 2018 from the surface of the Arctic Red during the ice break up are generally ^14^C enriched (mean F^14^C = 0.96 ± 0.01, *n* = 8), but the final sample on 29 May 2019 was more ^14^C depleted (F^14^C = 0.89 ± 0.01). In June 2019, DOC‐F^14^C values were similar to the other years in the data set (Figure 2). Overall, although annually collected “snap shot” water samples cannot resolve the duration of this export event, we note that June 2018 samples were not associated with anomalous discharge conditions and were collected during similar conditions as those from 2003 to 2019.

### 4.2 Aged DOC From Permafrost Soils and Climatic Warming

The large change in DOC‐F^14^C values in June 2018 can be viewed in the context of DOC transfer from soil to streams and in particular the delivery of previously stored, old DOC and weathering products to the hydrological network. Hydrological networks can access a much larger area than other potential causes of DOC aging, such as anthropogenic activity, erosional slumping, and wildfires (Butman et al., 2015; Gibson et al., 2018; Kettridge et al., 2012; Kokelj et al., 2013, 2015) (see supporting information discussion). The annual summer and freezing temperatures play important roles in the dynamics and thickness of the active layer (Kokelj & Burn, 2005). Air temperature records from Inuvik and Norman Wells during the freezing season (onset and the end of continuous freezing) show temperature increases over the last 76 years (Bush & Lemmen, 2019) (Figures S6, S7, and Table S4) and a pronounced temperature anomaly in winter 2017/2018. The winter 2018/2019 period shows an even higher temperature anomaly. In addition, we note an anomalous warm summer period in 2017 which was followed by colder summer seasons in 2018 and 2019 (Figure S8).

Observations of permafrost temperature in the northern Mackenzie River basin point to a warming of 0.5°C to 0.9°C and a thickening of the active layer by about 10% since 2000 (Biskaborn et al., 2019; Bush & Lemmen, 2019; Smith et al., 2018). The shift of the permafrost table due to thaw during the summer months occurs both vertically and laterally and allows the development of thin, perennial taliks within and above permafrost (Lamontagne‐Hallé et al., 2018; Walvoord et al., 2012; Zhang et al., 2008). Mild winters are insufficiently cold to completely counterbalance this thaw. Increased hydrogeologic connectivity in the fall enhances the drainage of surface soils (Liljedahl et al., 2016), the reduction of soil moisture, and the temporary increase of the water storage capacity (McCartney et al., 2006; Quinton et al., 2005; Teufel & Sushama, 2019). As a consequence, organic soils can be undersaturated prior to freeze back in fall. Under this regime, a larger portion of meltwater can infiltrate soils in spring, rather than entering streams as surface runoff (Teufel & Sushama, 2019). Percolating meltwater supplies sensible heat to the soil leading to the thaw of the upper permafrost table and the expansion of hydrologically connected pathways (Connon et al., 2018; Teufel & Sushama, 2019; Walvoord et al., 2019). Organic‐rich material concentrated below the active layer can be mobilized by this subsurface flow of water, mixed with meltwater, and subsequently transferred to streams (Plaza et al., 2019; Walvoord & Striegl, 2007; Walvoord et al., 2019). Supra‐permafrost taliks are expected to become more pronounced with continued warming, as a result of delayed active layer freeze up in fall accompanied by a poor freeze back in warm winters (Euskirchen et al., 2006; Serreze et al., 2000; Walvoord et al., 2019).

To better constrain the mechanisms operating in June 2018, we examine dissolved ions that originate from mineral weathering and organic matter cycling. We find that June 2018 waters exhibit higher abundances of calcium ions relative to Cl^−^ ions, a conservative tracer, and enhanced relative abundances of NO_3_^−^ ions (Figure 3a). In addition, DOC F^14^C values were negatively correlated with [NO_3_^−^] at all locations (Figure 3b). An increase input of NO_3_^−^ to high‐latitude streams, rivers, and lakes has been associated with gradual warming (e.g., Fouché et al., 2020; Frey et al., 2007; Harms & Jones, 2012; Jones et al., 2005; McClelland et al., 2007; Walvoord & Striegl, 2007), the draining of thermokarst environments (Abbott et al., 2015), and the more frequent occurrence of wildfires (Petrone et al., 2007). However, the fate of solute transfer from permafrost soils to streams depends on abiotic and biotic processes, complex interactions within the soil matrix, and residence times (Harms & Jones, 2012; Spencer et al., 2015; Striegl et al., 2005). While Ca^2 +^ ions are derived from carbonate weathering in the Mackenzie River basin (Tank et al., 2016), NO_3_^−^ is produced by nitrification following organic matter mineralization and the dieback of plant roots (Keuper et al., 2012; Treat et al., 2016). In fall and winter, NO_3_^−^ can accumulate as a result of continued microbial remineralization, while the uptake by plants is reduced due to dormancy (Edwards & Jefferies, 2013; Treat et al., 2016). Extractions performed on permafrost soils show that both Ca^2 +^ and NO_3_^−^ ions can be immobilized by freezing and enriched relative to other ions just below the active layer (Kokelj & Burn, 2005; Reyes & Lougheed, 2015). The release of aged DOC in 2018 was accompanied by a doubling in [NO_3_^−^] (Figure 3b and Table S3). This suggests that waters accessing and routed through soils near the permafrost table serve as a viable source of aged DOC in June 2018.

Although the winter 2018/19 experienced record warmth, we observe no aged DOC in the June 2019 samples. However, preceding summer temperatures in 2018 are lower than the long‐term average. Indeed, we find that June 2019 waters have lower [NO_3_^−^]/[Ca^2 +^] ratios and similar [NO_3_^−^] as June samples from 2003–2017 (Figure 3a). The annual thaw depth strongly varies as a function of near‐surface temperature (Kokelj & Burn, 2005; Teufel & Sushama, 2019). Given the cooler summer in 2018, it is possible that the annual thaw was less severe and likely supported the formation of soil ice that counteracted the development of taliks and hydraulically connected pathways in the following spring 2019. Another explanation could be that our sampling missed an aged DOC signature that occurred earlier or later in 2019 (Figure 1c). Alternatively, it could reflect an exhaustion of DOC first mobilized in June 2018 and/or concurrent increases in mobilization of young surface organic carbon (Dean et al., 2018; Feng et al., 2013). Differences in the contributions from specific higher‐order streams and/or the biodegradability of DOC during transport (Holmes et al., 2008; Mann et al., 2015; Vonk et al., 2013) could have also played a role.

While it is not possible from our data set to unravel the details of carbon mobilization in permafrost zones undergoing thaw, our work highlights the potential transitory nature of aged DOC export events. Despite the challenges of long‐term sampling of river carbon species and coupled analyses of ^14^C activity, our observations underline the potential for rapid and large‐scale mobilization of aged carbon pools previously sequestered in Arctic permafrost soils.

## 5 Conclusions

In this study, radiocarbon measurements of DOC in the Mackenzie River and its two large northern tributaries (Peel and Arctic Red Rivers) from 2017 to 2019 are combined with previously published data to explore interannual variability in DOC export from a major Arctic fluvial system. DOC ages from 2003 to 2017 suggest a predominant source from recent vegetation and soils. However, samples from June 2018 record a previously undocumented and widespread export of aged DOC, before returning to modern F^14^C values in June 2019. This aged DOC was accompanied by high [NO_3_^−^] in all three rivers. The change in DOC age and solute flux that we observe appears consistent with the formation of supra‐permafrost taliks and the deeper percolation of groundwater, resulting in mobilization of organic matter and weathering products preserved in previously frozen, organic‐rich soil horizons. Limitations in sampling coverage preclude elucidation of the magnitude and dynamics of aged DOC export and associated carbon cycle feedbacks in response to a warming climate and highlight the need for sustained, long‐term observations and refined sampling and measurement strategies. Irrespective of these caveats, our observations reveal the potential for abrupt and pervasive mobilization of hitherto sequestered carbon from vast permafrost regions of the Arctic.

## Conflict of Interest

The authors declare no conflict of interest.

## Supporting information

Supporting Information S1

Table S1

## Data Availability

Data from 2003 to 2016 are available in the ArcticGRO repository (https://arcticgreatrivers.org/data/). All data generated from 2017 to 2019 are openly available in the EarthChem Library (https://doi.org/10.26022/IEDA/111725). A detailed method description and supporting information figures and tables can be found in the supporting information. Correspondence and requests for materials should be addressed to M. S. S. (melissa.schwab@erdw.ethz.ch).
