# Supplementary material for: An Abrupt Aging of Dissolved Organic Carbon in Large Arctic Rivers
Source: Geophys Res Lett. 2020 Dec 8;47(23):e2020GL088823. doi: 10.1029/2020GL088823 (PMC7757186; doi:10.1029/2020GL088823)
Supplement: Supplementary file 1 — Supporting Information S1 [file GRL-47-e2020GL088823-s001.pdf]

Geophysical Research Letters

Supporting Information for

An Abrupt Aging of Dissolved Organic Carbon in Large Arctic Rivers

M. S. Schwab[1*], R. G. Hilton[2], P. A. Raymond[3], N. Haghipour[1,4], E. Amos[5], S. E. Tank[6], R. M. Holmes[7], E. T. Tipper[8] and T. I. Eglinton[1]

[1]Department of Earth Sciences, ETH Zurich, Zurich, Switzerland.

[2]Department of Geography, Durham University, Durham, UK.

[3]School of Forestry and Environmental Studies, Yale University, New Haven, CT, USA.

[4]Laboratory of Ion Beam Physics, ETH Zurich, Zurich, Switzerland

[5]Aurora Research Institute, Inuvik, NT, Canada.

[6]Department of Biological Sciences, University of Alberta, Edmonton, AB, Canada

[7]The Woods Hole Research Center, Falmouth, MA, USA.

[8]Department of Earth Sciences, University of Cambridge, Cambridge, UK.

Contents of this file

Text S1 to S3
Figures S1 to S10
Tables S1 to S6

Additional Supporting Information (Files uploaded separately)

Table S1. River suspended dissolved and particulate material.

Introduction

This supporting information contains procedures comprising sample collection, the analyses of dissolved and particulate organic carbon as well as major ions. A detailed description of applied statistical methods and calculations are provided. Geochemical and statistical results are described and listed below. We further describe potential correlations between discharge, DOC

concentration, DOC-F^{14}C and major ions. In **Section S3** we discuss physical mechanism which could influence the water chemistry of Arctic streams and rivers including anthropogenic pollution, wildfires and erosional events.

S1. Methods

Samples from 2003 to 2012 were collected by the Pan-Arctic River Transport of Nutrients, Organic Matter, and Suspended Sediments (PARTNERS) and Arctic Great Rivers Observatory (ArcticGRO) projects as described at www.arcticgreatrivers.org. Detailed sample collection information can be found in Raymond et al. (2007) and Holmes et al. (2012). In summary, river water was collected using a 60-kg depth-integrating sampler (model US D-96) fitted with a Teflon nozzle and Teflon bags. One sample consists of a mixture of five depth-integrated transects across the channel. Samples collected from 2013 to 2016 consist solely of surface samples. Samples were filtered through a 47-mm-diameter quartz filter (Whatman QMA; 0.7 μm) into an acid-leached 500 mL polycarbonate bottle and stored frozen until further analysis. Major ions were directly filtered into acid leached HDPE bottles where cation samples were immediately preserved with HCl to pH 2-3 (Holmes et al., 2012; Tank et al., 2012).

We collected water from river depth-profiles during the peak freshets in June 2017, 2018 and 2019 as per methods outlined by Hilton et al. (2015). Two or more transects with an Acoustic Doppler Current Profiler (2017-18: RioGrande, Teledyne; 2019: RiverRay, Teledyne) were used to measure channel depth and water velocity, and subsequently to determine instantaneous water discharge. For every sampling location (**Figure 1a**), we chose a sampling point with the maximum depth and the highest backscatter corresponding to the highest turbidity. A modified horizontally-mounted ~5.1 L Niskin bottle allowed the recovery of water from different depths in order to compensate for hydrodynamic sorting and transport of particles within the river. The collected water was transferred to a sterilized plastic bag (Jigsaw Bag in Box LTD), and the sample volume was determined by weighing the river water sample. Within 48 hours, the samples were subsequently filtered using pre-cleaned filtration units through 142 mm diameter 0.22 μm polyethersulfone filters. The exact same filtration units were used in 2017 and 2018. In 2019, steel filtration units with Teflon linings were utilized. Filtered water samples for dissolved organic carbon (DOC) analysis were collected in 120 mL pre-combusted (450°C for 6 hours) amber glass bottles, and then acidified with 85% H_3PO_4 (120 μL) to pH~2. DOC samples were stored and transported dark and cooled (4°C) until analysis. Water sample aliquots were collected for ion analysis in pre-cleaned HDPE bottles, and acidified with trace analysis grade HNO_3 to pH~2 for cation aliquot and left unacidified for the anion aliquot. After filtration, the filter was folded, placed in a pre-combusted aluminum foil envelop, immediately frozen and kept frozen until freeze drying.

During the course of the ice break-up in May 2018, 8 water samples (~ 2 L) were collected from the Arctic Red River in acid cleaned 1 L Teflon bottles and stored dark and cooled (4°C). The water was processed during the field campaign as described above.

Geochemical analyses

DOC concentrations of samples collected by the PARTNERS and ArcticGRO projects were analyzed using a Shimadzu TOC analyzer. The UV light oxidation method was performed converting DOC to CO_2. The CO_2 was trapped and cryogenically purified at Yale University (see Raymond et al., 2007; Barnes et al., 2018). Carbon isotopes were analyzed at the National Ocean

Sciences Atomic Mass Spectrometry (NOSAMS) facility at the Woods Hole Oceanographic Institution or at the University of Arizona Accelerator Mass Spectrometer (AMS) facility.

DOC concentrations for the present study (2017-2019) were determined using a Shimadzu system (TOC-L Series) at the Department of Environmental System Science at ETH. DOC-^{14}C measurements were performed according to the method described by Lang et al. (2012, 2016). In brief, a 5 mL aliquot of each sample (corresponding between 10 to 53 μgC) was transferred into pre-combusted 12-mL Exetainer screw-capped vials with a butyl rubber septum (Labco, Buckinghamshire, UK, P/N 938W). To first remove inorganic carbon, 1 mL of acidified sodium persulfate solution (100 mL H_2O + 4.0 g $Na_2S_2O_8$ + 200 μL of 85% H_3PO_4) was added to the sample, and the mixture was then capped and purged with high-purity helium gas (Grade 5.0, 99.9999% pure, for 10 min) to drive off CO_2. Subsequently, the samples were heated at 100°C for 1 hour, converting DOC to CO_2. ^{14}C measurements of the produced CO_2 were performed using a mini carbon dating accelerator mass spectrometer (MICADAS AMS) system equipped with a gas ion source (GIS) at the Laboratory for Ion Beam Physics (LIP) at ETH Zurich. Sucrose (Sigma, $\delta^{13}C$ = -12.4‰ VPDB, $F^{14}C$ = 1.053 ± 0.003) and phthalic acid (Sigma, $\delta^{13}C$ = -33.6‰ VPDB, $F^{14}C$ < 0.0025) were used to assess blank contribution. Typical sample sizes ranged between 10 to 53 μgC. The contribution of extraneous carbon evaluated using a model of constant contamination described in Haghipour et al. (2019) amounted to ~1 μgC.

Suspended sediment samples from 2017 and 2018 were freeze-dried and ground with mortar and pestle. Samples (~30 mg, ~200-300 μgC) were vapor acid-treated to remove inorganic carbon (Bao et al., 2018), similar to methods applied to 2010 and 2011 samples reported by Hilton et al. (2015). Bulk OC concentration measurement was performed using an elemental analyzer isotope ratio mass spectrometer (EA-IRMS; LIP, ETH Zurich). Radiocarbon analysis of the samples was measured directly as CO_2 gas using an EA-AMS MICADAS system (LIP, ETH Zurich; Wacker et al., 2010). The samples were calibrated against Oxalic Acid II (NIST SRM 4990C) as well as an in-house soil and shale standard. All samples were corrected for constant contamination (3-5 μgC) (Haghipour et al., 2019; Welte et al., 2018).

Major cations and anions were analyzed for samples spanning the period from 2003 to 2016 at the Woods Hole Research Center using a Dionex Ion Chromatography system (GP50, AS50 and ED50; Dionex Corp.). Samples from 2017-2018 were measured at Durham University, and samples from 2019 at ETH Zurich using a Dionex Ion Chromatography system (DX-120, Thermoscientific). Anions were eluted (25 μL, 1.4 mL/min) with carbonate/hydrogencarbonate solution through an IonPac AS14,4 analytical column using ASRS-Ultra II suppressor. Cations (25 μL, 1.3 mL/min) were analyzed using a IonPac CS12A exchange column and a CSRS-Ultra Suppressor with methansulfonic acid as eluent. Analytical reproducibility of the geochemical analysis was 5%.

Analytical precision and reproducibility

All DOC samples measured in this study were collected and prepared by M.S.S. and measured by N.H. Pre-combusted amber vials were rinsed three times before the collection of the filtrate. The filtrate was acidified with ultrapure HNO_3, capped (Teflon-lined caps), tapped with Teflon tape, stored and transported cooled (4°C) and dark until analysis. During each field campaign, samples were treated with the identical acid, thus, particular attention was placed on the single use of pipette tips to avoid cross-contamination.

Per campaign, DOC samples were analyzed in at least two different batches alongside standards (phthalic acid, sucrose), the utilized oxidizing agent ($Na_2S_2O_8$), and processing blanks. Batches

containing samples from 2018 showed aged as well as modern DOC-^{14}C values (0.33 to 1.06). Moreover, replicates for the samples CAN18-02, CAN18-12, CAN18-31, and CAN18-32 (**Table S1**) were reproduced well within error. Based on low contamination values and the high reproducibility of our results, we reject possible errors during analyses.

Statistical analyses

All statistical analyses were realized using RStudio (version 1.2. © 2009–2019 RStudio, Inc). Linear correlation between two variables are reported as Pearson's product-moment correlation coefficients (r) and significance *p*-values. Statistically significant differences between medians for different years for each river system were tested separately using non-parametric Wilcox and Kruskal-Wallis rank sum tests. If significant between-group differences were identified, we conducted multiple comparisons using Dunn's test and a Benjamini–Hochberg adjustment. All statistical comparisons are reported at the 95% confidence interval ($p < 0.05$).

Calculation of air temperature anomalies

Daily temperature means and precipitation data were obtained from the Environment Canada website (http://climate.weather.gc.ca/). Where possible, temperature data were retrieved from one continuous station. However, changes in instrumentation and relocation of observing sites introduced uncertainties to the data set which were not accounted or corrected for due to the paucity of nearby acceptable stations (e.g., the long-term *Inuvik A* station was abandoned and replaced by *Inuvik Climate* at a higher elevation). The data sets were explored, cleaned and prepared for analysis. Missing values up to 10 days were interpolated using the na.interpolation ("stine") function of the R package imputeTS. Freezing periods with more than ten following days of missing data were excluded from further processing. We averaged temperature values over the duration of freezing, which is defined as the cumulative days since the start of continuous freezing (**Figure S6, Table S4**). We define the end of the freezing period with seven days of consecutive days above 0°C. Temperature anomalies were calculated relative to the 30-year period from 1961 to 1990. A 5-year rolling average was calculated with the R package zoo (rollmean).

S2. Results

Particulate organic carbon

The POC-F^{14}C values vary between 0.28 and 0.64, with no systematic shifts between years. The ^{14}C-depletion of particulates in Mackenzie River at Tsiigehtchic the Arctic Red and the Peel River has been previously recognized (Goñi et al., 2005; Hilton et al., 2015) and attributed to a mixture of aged organic matter from peat and permafrost soils (~70% of the mass) with rock organic carbon (Hilton et al., 2015).

Major ions

The correlation between discharge, DOC concentration, DOC-F^{14}C and major ion concentration are depicted for the Mackenzie River at Tsiigehtchic in **Figure S9** and **Table S5**. The relationship between discharge and the base weathering cations Na^+, K^+, Mg^{2+} and Ca^{2+} and anions NO_3^-, SO_4^{2-} and Cl^- show clear dilution effects during periods of increased flow. Correlation coefficients range from -0.57 to -0.94. DOC is positive correlated with discharge, while DOC-F^{14}C indicates no association with discharge. DOC concentrations are negatively related to Na^+, Mg^{2+}, Ca^{2+},

NO_3^-, SO_4^{2-} and Cl^-, but no correlation is displayed for K^+ and DOC-$F^{14}C$. DOC-$F^{14}C$ does not exhibit any strong trends with discharge or ions. Major cations and anions show positive correlation with each other, suggesting a similar mobilization and mechanism of delivery. Meaningful exceptions are $NO_3^-$and SO_4^{2-}, $NO_3^-$and K^+, and NO_3^- and Ca^{2+}. The lack of correlation highlights the differences in origin and transport of the biologically active NO_3^- and the weathering ions SO_4^{2-}, Mg^{2+}, and Ca^{2+} which are produced during carbonate dissolution.

S3. Potential mechanisms for aged DOC input

Although anthropogenic activity can enhance delivery of aged DOC by deforestation and land use change (Drake et al., 2019; Moore et al., 2013), or by release of wastewater and petroleum products (Butman et al., 2015; Regnier et al., 2013), it is difficult to ascribe such processes as a factor in the Mackenzie River due to low population densities (Butman et al., 2015). Hydrocarbon extraction does take place in the upper Mackenzie Basin (Hein, 2006; Snowdon et al., 1987) but not in the Arctic Red and Peel basins, which have the most ^{14}C-depleted signature in June 2018 (**Figure 2**). In glacial settings, atmospheric aerosols have been invoked to explain aged DOC (Hood et al., 2009; Singer et al., 2012; Stibal et al., 2012). However, only ~0.13% of the Mackenzie River basins is glaciated (https://open.canada.ca/data), and deposition of carbon-rich aerosols is insignificant relative to natural DOC sources in the basin (Vonk et al., 2015), and incompatible with one-time mobilization of these materials.

Wildfires are an essential component of the circumboreal forest (Flannigan et al., 2009; Turetsky et al., 2004), and impact the ecohydrological function of permafrost zones (Kettridge et al., 2012) by enhancing thermal conductivity (Brown et al., 2015), improving drainage to subjacent mineral soils (Hinzman et al., 2003), expansion of taliks (Gibson et al., 2018; Kettridge et al., 2012), deepening of the active layer (Brown et al., 2015; Walvoord et al., 2019) and the formation of thermokarst bogs (Gibson et al., 2018). Fire-related thaw also enhances streamflow (Quinton et al., 2011; Tank et al., 2016), and export of DOC and nutrients (Olefeldt et al., 2014; Tank et al., 2016). However, annual fire activity affects less than 1% of the Mackenzie River catchment (https://cwfis.cfs.nrcan.gc.ca) and is mainly focused to the southern part of the basin, with very low fire occurrence in the Arctic Red basin where the most ^{14}C-depleted DOC was observed (**Figure 1, Table S6**). It is therefore difficult to invoke a specific role of wildfire to explain the June 2018 DOC measurements.

Erosional events such as a permafrost slumping, enhanced bank erosion (Kokelj et al., 2017; Vonk et al., 2015) or larger-scale mega-slumps (Kokelj et al., 2013, 2015) could contribute to the ^{14}C depletion of the DOC pool observed in 2018. Erosional events are known to be sources of ^{14}C-depleted POC to rivers (Goñi et al., 2005, 2013; Hilton et al., 2015; McClelland et al., 2016). However, the aged DOC in June 2018 is not accompanied by a change in POC concentration, nor its ^{14}C activity (**Figure S10**). The export of aged POC has been a persistent feature of the Mackenzie river for several decades (Goñi et al., 2005; Hilton et al., 2015; Vonk et al., 2019), whereas – with the exception of 2018, DOC has remained 'young' (**Figure 2**). These observations, and the fact that three separate catchment areas record old DOC in June 2018, argue against changes in erosion behavior of solid material as a cause for the large shifts in DOC-$F^{14}C$ values.

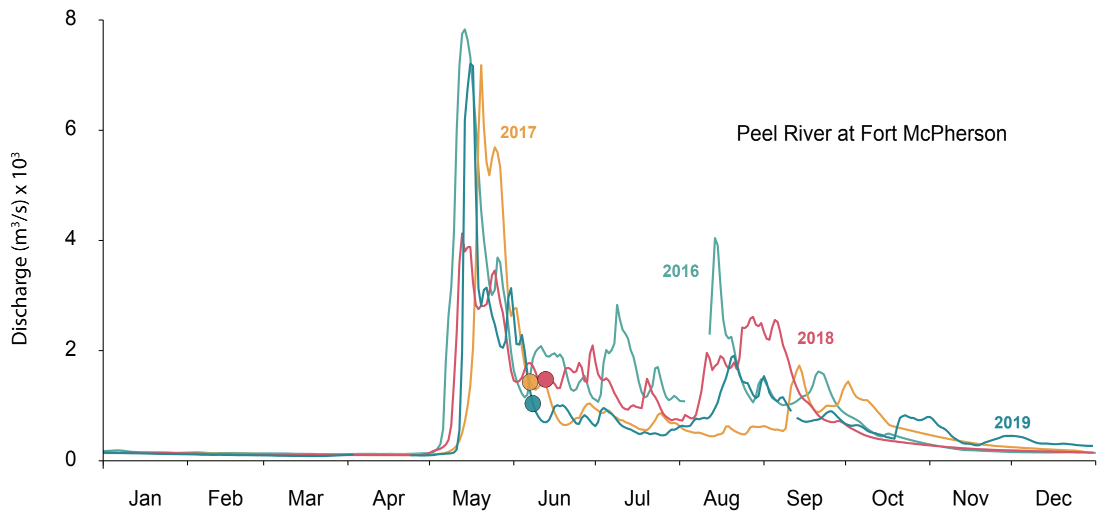

Figure S1. Discharge at the Peel River at Fort McPherson from 2016 to 2019 (HYDAT, http://www.wateroffice.ec.gc.ca). Dots denote sampling dates.

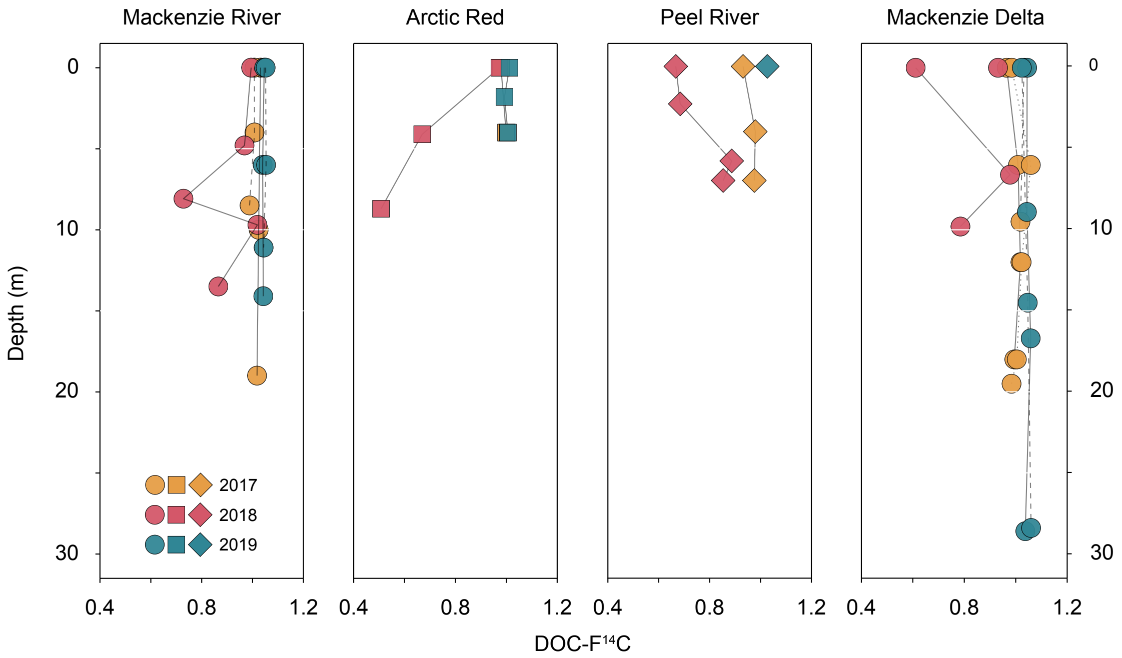

Figure S2. Depth variations in radiocarbon activity ($F^{14}C$) of DOC in the water column. River depth profiles for the Mackenzie River at Tsiigehtchic and the Mackenzie Delta (circles), the Arctic Red (squares) and the Peel River (diamonds) color coded for different years.

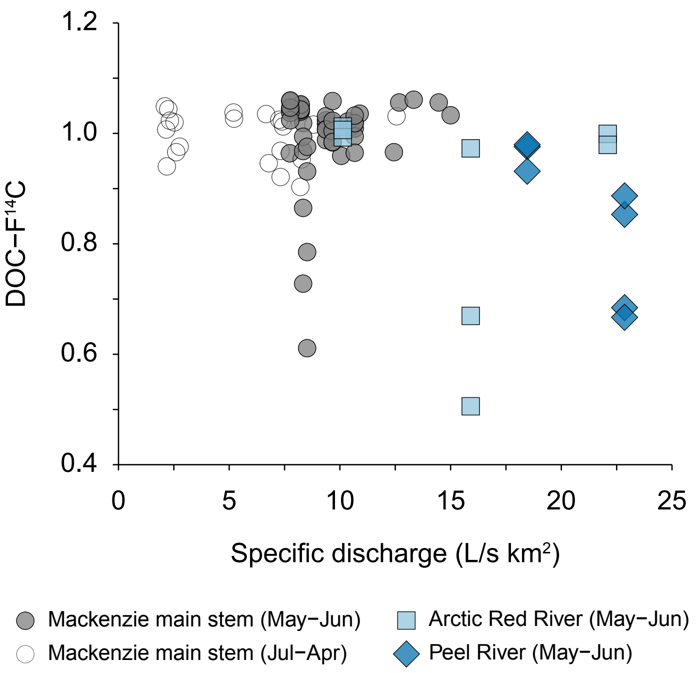

Figure S3. The radiocarbon activity ($F^{14}C$) of DOC plotted against the specific discharge (L/s km^2). Circles represent samples from the Mackenzie River at Tsiigehtchic and the delta, with filled circles samples collected close to ice break up and peak water discharge from 2003 to 2019. Samples from the Arctic Red (light blue square) and Peel River (dark blue diamond) are also shown for 2017-2019.

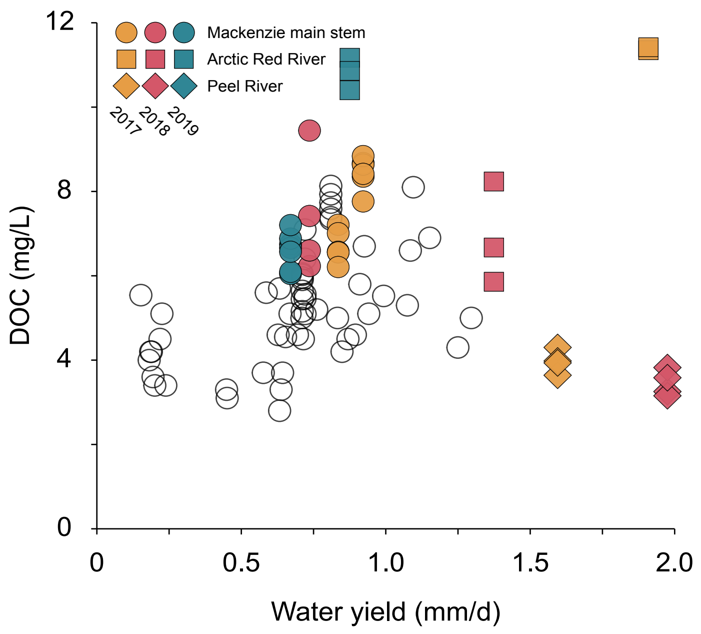

Figure S4. DOC concentrations (mg/L) as function of water yield: Mackenzie main stem (circles), the Arctic Red (squares), the Peel River (diamonds). Open circles represent the

ArcticGRO data set from the Mackenzie River at Tsiigehtchic from 2003 to 2013. Symbols are color coded for different years.

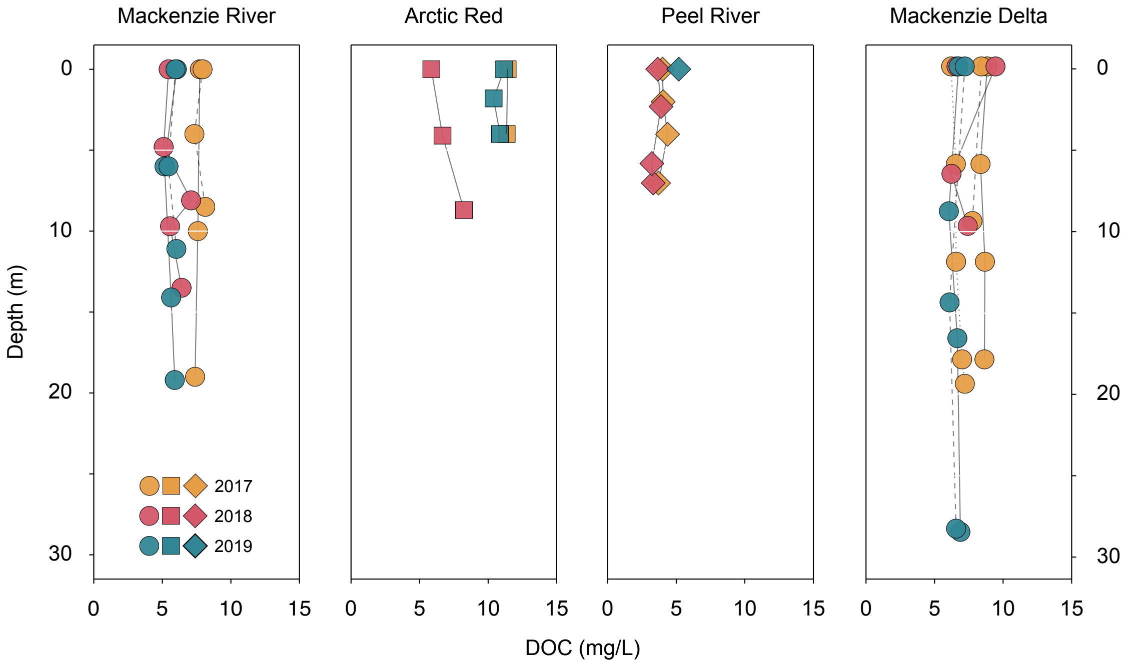

Figure S5. Depth variations in DOC concentrations (mg/L) in the water column. River depth profiles for the Mackenzie River at Tsiigehtchic and the Mackenzie Delta (circles), the Arctic Red (squares) and the Peel River (diamonds) color coded for different years.

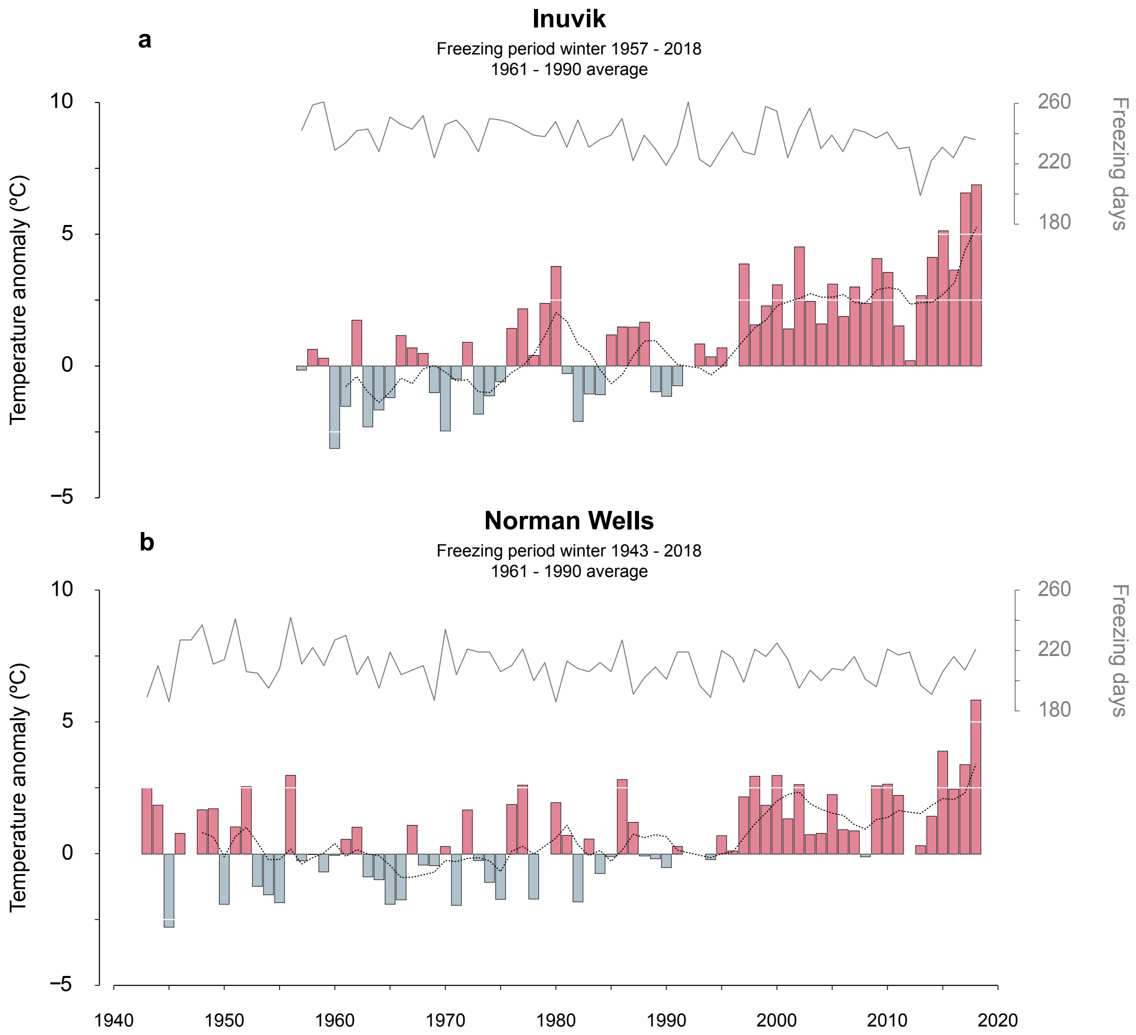

Figure S6. Average air temperature anomalies and freezing days for the freezing period from 1943 to 2019 for Inuvik (**a**) and Norman Wells (**b**). The baseline is defined as the mean over the 1961 - 1990 reference period. The freezing period begins with continuous freezing and ends with seven consecutive days above 0. The black line indicates a five-year mean (rolling average). Source: Environment Canada (http://climate.weather.gc.ca/).

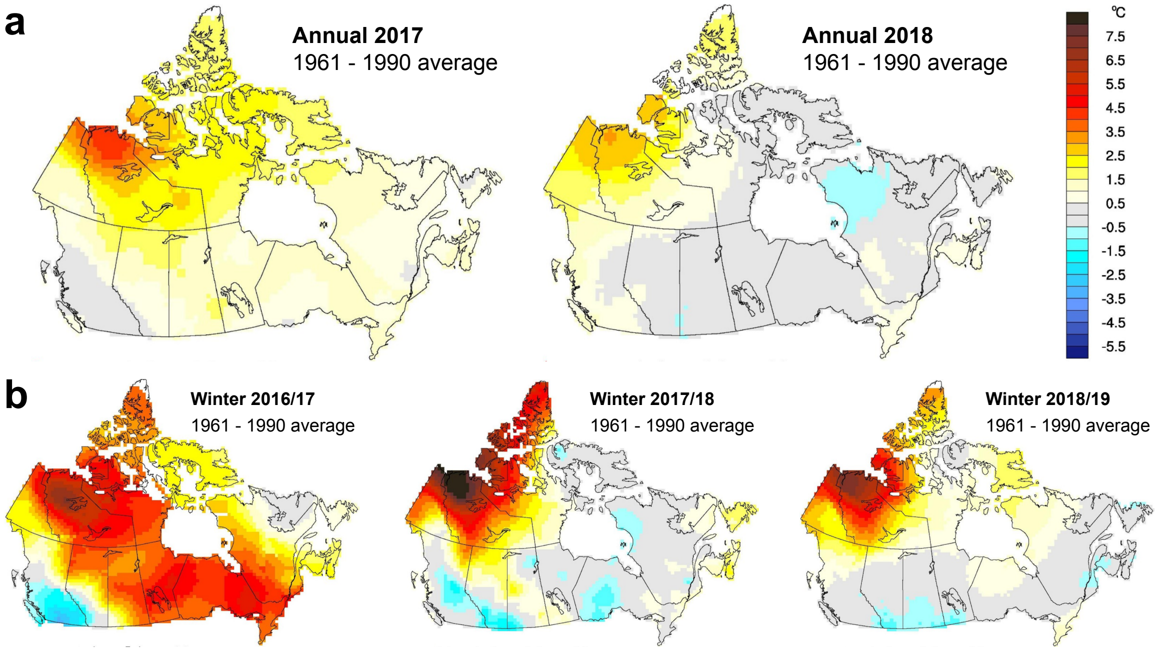

Figure S7. Temperature anomalies. **a**, Annual temperature anomalies showing 3.0 to 4.5°C temperature increase above average baseline in the upper Mackenzie River basin. **b**, In comparison to the baseline, the Mackenzie River basin experienced increased temperatures between 4.5 to 8.0°C in winter. CTVB, Environment Canada (https://www.canada.ca/en/environment-climate-change/services/climate-change/science-research-data/climate-trends-variability/trends-variations.html).

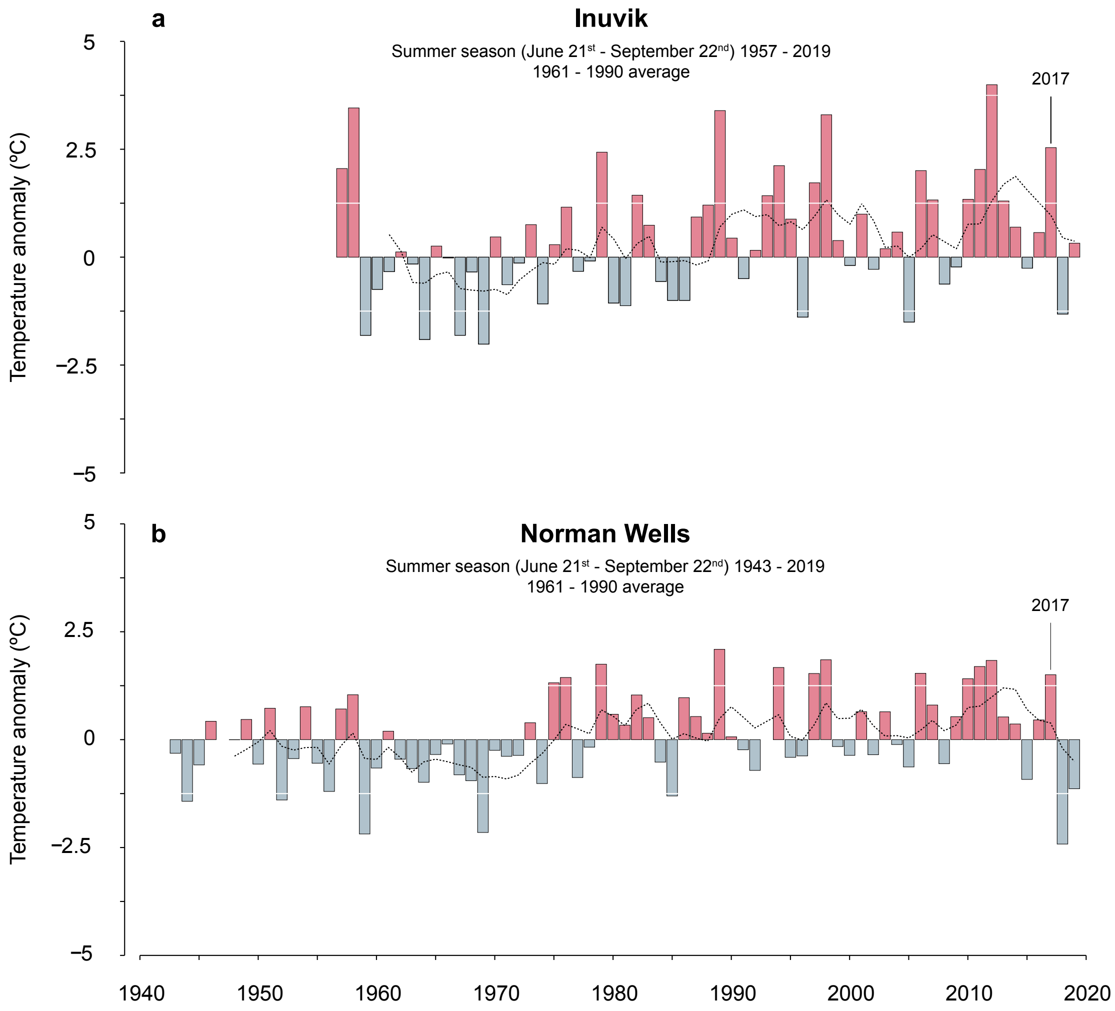

Figure S8. Average air temperature anomalies for the summer period from 1943 to 2019 for Inuvik (**a**) and Norman Wells (**b**). The baseline is defined as the mean over the 1961 - 1990 reference period. The summer seasons refers to the period from June 21st to September 22nd. The black line indicates a five-year mean (rolling average). Source: Environment Canada (http://climate.weather.gc.ca/).

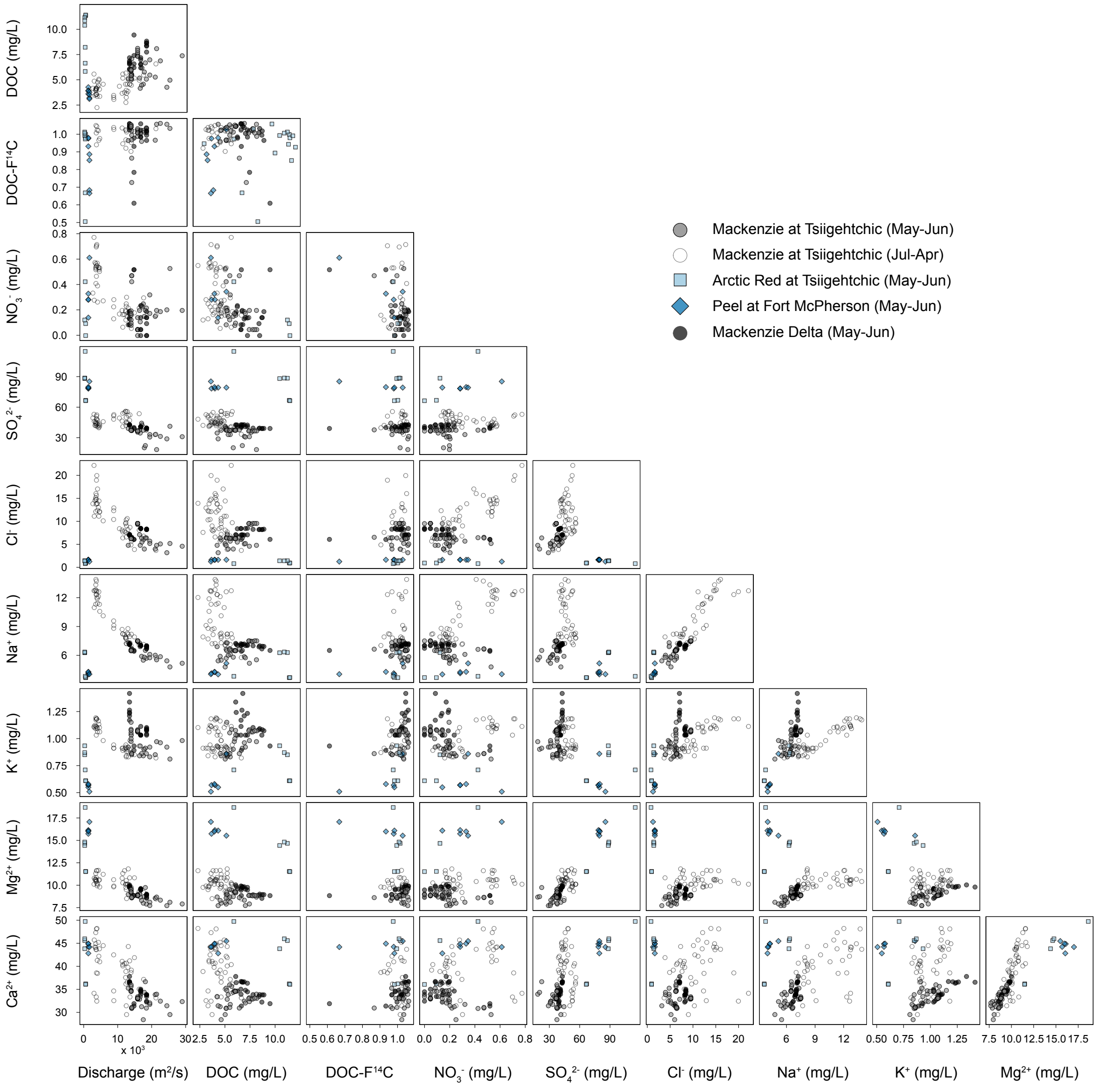

Figure S9. Bi-variate relationships among discharge, DOC, DOC-F^{14}C and major ions. Circles are samples from the Mackenzie River at Tsiigehtchic (grey) and the main channel in the delta (black), with filled circles samples collected in May and June. Samples from the Arctic Red (light blue square) and Peel Rivers (dark blue diamond) are also shown for 2017-2019. Data from 2003 to 2016 are provided by the ArcticGRO database.

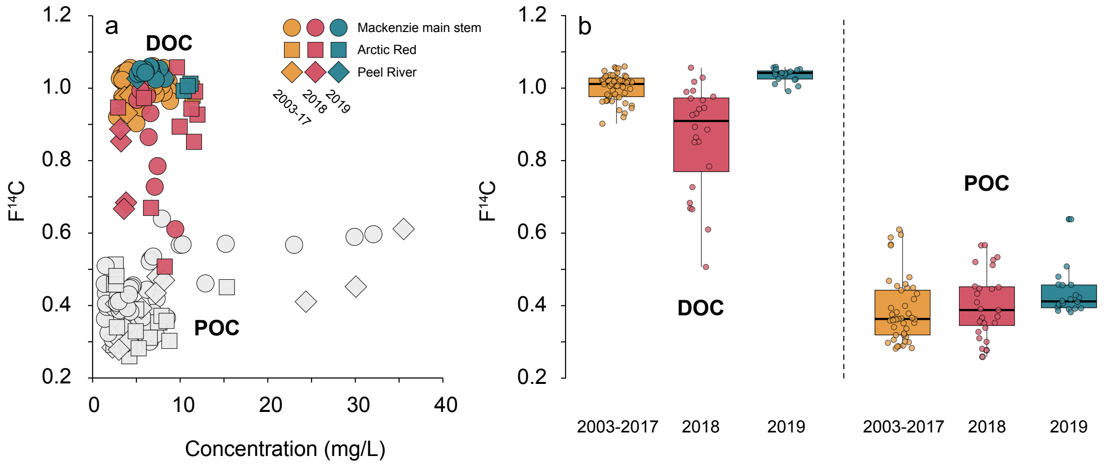

Figure S10. DOC and POC radiocarbon activity ($F^{14}C$) in the Mackenzie River: **a**, $F^{14}C$ versus concentration (mg/L), where colored symbols indicate dissolved and grey symbols indicate particulate organic carbon. Circles represent the Mackenzie main stem, square the Arctic Red and diamonds the Peel River, respectively. **b**, Boxplot of radiocarbon activity ($F^{14}C$) of DOC and POC for different years, with the median (black line), first and third quartile (box) and confidence interval (lines) shown. The POC data set includes published values from 2010 and 2011 (Hilton et al., 2015).

Table S1. River suspended dissolved and particulate material.

	Year	n	Mean		Kruskal-Wallis rank sum test			Dunn's Test	
			M	SD	df	χ^2	Sig.	contrasts	Benjamini - Hochberg
Mackenzie River at Tsiigehtchic	2003-2017	39	1.005	0.006	2, 47	13.95	**< 0.001**	2018 - 2003-17	**0.027**
	2018	5	0.915	0.054				2018 - 2019	**< 0.001**
	2019	6	1.046	0.002				2003-17 - 2019	**< 0.003**
Arctic Red at Tsiigehtchic	2017	2	0.989	0.010	2, 13	3.74	0.15		
	2018	11	0.890	0.049					
	2019	3	1.004	0.006					
Mackenzie Delta	2017	11	1.008	0.008	2, 21	15.57	**< 0.001**	2018 - 2017	**0.025**
	2018	4	0.826	0.083				2018 - 2019	**< 0.001**
	2019	9	1.044	0.004				2017 - 2019	**0.008**
					**Wilcox rank sum test**				
					W	Sig.			
Peel River at Fort McPherson	2017	3	0.962	0.015	12	0.057			
	2018	4	0.773	0.057					

Table S2. Non-parametric one-way analysis of variance.

Sample ID	$[Na^+]$ µmol	$[K^+]$ µmol	$[Mg^{2+}]$ µmol	$[Ca^{2+}]$ µmol	$[Cl^-]$ µmol	$[NO_3^-]$ µmol	$[SO_4^{2-}]$ µmol
CAN17-01	299.3	27.6	364.5	833.4	231.6	2.1	408.2
CAN17-02	301.9	27.6	368.2	842.4	231.9	2.9	408.2
CAN17-03	290.1	26.3	351.0	803.4	231.3	2.9	407.6
CAN17-04	301.0	27.4	367.8	842.1	233.8	3.6	409.2
CAN17-05-08	304.0	28.1	367.4	843.4	233.0		412.0
CAN17-09	292.7	26.6	355.5	808.4	235.0	0.7	413.2
CAN17-10	304.0	27.9	370.3	843.6	232.4	0.7	412.0
CAN17-12	326.2	27.6	365.8	826.6	269.4	1.4	384.2
CAN17-13	323.6	27.1	363.3	820.9	268.2	1.4	381.7
CAN17-14	324.5	27.1	363.3	820.2	262.0		370.8
CAN17-15	326.2	27.4	368.2	832.1	268.0	0.7	382.3
CAN17-16	324.1	27.1	362.5	819.9	269.1	1.4	383.9
CAN17-17-20	326.4	27.4	364.6	824.5	268.7	1.6	383.1
CAN17-21	326.2	27.4	363.7	822.4	268.5	2.9	382.3
CAN17-22	326.7	27.4	364.5	824.6	269.1	1.4	383.3
CAN17-26-29	158.6	15.4	467.0	888.0	27.7	2.4	673.1
CAN17-30	160.9	15.6	473.2	898.5	27.4	0.7	690.8
CAN17-33-36	177.3	14.3	643.9	1088.9	47.2	3.9	816.3
CAN17-37	175.3	14.1	660.4	1066.2	47.1	2.9	824.9
CAN17-38	185.3	14.6	662.4	1116.8	46.8	5.0	815.5
CAN17-39	186.6	14.8	656.2	1118.3	47.1	5.7	829.6
CAN17-46-49	305.4	26.3	389.2	858.8	239.5	0.7	428.5
CAN17-50	304.9	26.3	392.9	867.1	238.1	2.9	426.9
CAN17-51	304.5	28.4	388.4	845.4	238.1	1.4	426.6
CAN17-52	307.1	26.3	395.8	872.5	237.8	1.4	426.0
CAN17-53	306.2	26.3	394.2	862.8	236.7	4.3	425.1
CAN18-02	283.2	23.8	363.3	794.9	171.8	8.6	408.5
CAN18-02 rep							
CAN18-03							
CAN18-04							
CAN18-05-11							
CAN18-12							
CAN18-12 rep							
CAN18-14	279.3	22.8	363.7	782.0	170.9	8.6	418.2
CAN18-18							
CAN18-19-25							
CAN18-26	288.8	22.0	354.2	771.2	184.5	7.9	394.5
CAN18-28							
CAN18-29	288.0	21.7	353.0	769.0	181.9	7.1	391.4
CAN18-31	290.6	22.0	355.9	774.7	180.2	7.9	386.7
CAN18-31 rep							
CAN18-32							
CAN18-32 rep							
CAN18-36							
CAN18-37							
CAN18-38	166.6	18.2	766.1	1237.8	23.7	7.1	1192.9
CAN18-49							
CAN18-51							
CAN18-52							
CAN18-53							
CAN18-54	176.2	13.0	700.3	1100.1	36.1	10.0	887.3
CAN18-55							
CAN18-56							
CAN18-57							
CAN18-58							
CAN18-59							
CAN18-60							
CAN18-61							
CAN18-62							
CAN19-03	310.4	36.2	402.6	910.3	198.7	2.0	444.3
CAN19-04	315.1	34.1	412.4	941.4	200.6	3.3	445.8
CAN19-05	310.7	32.2	405.6	911.5	200.2	3.6	446.8
CAN19-06	312.1	30.9	403.7	912.4	197.8	2.7	447.3
CAN19-07	308.7	31.5	401.1	905.0	199.6	2.8	443.5
CAN19-08	311.1	31.7	404.9	908.3	198.1	3.6	444.6
CAN19-09	313.3	29.9	407.3	914.3	197.8	2.0	446.5
CAN19-14	224.9	22.0	637.3	1132.1	47.2	5.9	825.2
CAN19-19	282.2	26.4	376.3	861.7	177.2	3.1	394.5
CAN19-20	282.1	29.4	379.5	866.3	179.1	3.7	392.3
CAN19-21	284.6	27.7	389.2	887.9	177.5		393.3
CAN19-22	282.1	24.8	380.4	864.1	176.6	3.3	392.8
CAN19-23	282.7	25.5	381.1	861.6	178.6	1.9	391.3
CAN19-24	285.9	26.9	392.1	896.5	177.2	2.6	390.2
CAN19-25	281.7	27.5	380.1	860.7	175.1	2.2	389.0
CAN19-32	273.6	21.7	602.2	1135.0	41.2	2.6	921.3
CAN19-33	272.8	23.8	592.4	1090.9	40.8		917.2
CAN19-34	276.2	22.2	608.2	1144.4	40.5		922.6

Table S3. Riverine dissolved major anions and cations.

	Inuvik			Norman Wells		
Year	Mean freezing temperature (ºC)	Std. Deviation	Days of freezing	Mean freezing temperature (ºC)	Std. Deviation	Days of freezing
1943				-16.11	9.99	189
1944				-16.77	10.00	210
1945				-21.40	9.71	186
1946				-17.84	13.52	227
1947						227
1948				-16.95	13.47	237
1949				-16.91	10.47	211
1950				-20.53	11.70	214
1951				-17.60	13.46	241
1952				-16.07	11.90	206
1953				-19.86	10.81	205
1954				-20.17	10.45	195
1955				-20.47	12.75	208
1956				-15.64	11.95	242
1957	-19.34	12.62	242	-18.88	12.03	211
1958	-18.56	12.48	259	-18.65	11.42	222
1959	-18.89	13.14	261	-19.31	9.62	210
1960	-22.32	11.34	229	-18.68	11.17	227
1961	-20.72	11.14	234	-18.07	11.76	230
1962	-17.45	13.51	242	-17.61	12.41	204
1963	-21.50	12.76	243	-19.49	10.73	216
1964	-20.85	13.30	228	-19.60	13.68	195
1965	-20.39	13.55	251	-20.53	12.89	219
1966	-18.03	12.96	246	-20.37	11.30	204
1967	-18.50	12.08	243	-17.54	10.60	207
1968	-18.71	14.69	252	-19.05	13.70	210
1969	-20.20	11.89	224	-19.07	10.84	187
1970	-21.66	12.47	246	-18.34	13.59	234
1971	-19.68	12.04	249	-20.57	11.21	204
1972	-18.29	12.28	241	-16.95	12.16	221
1973	-21.01	10.94	228	-18.87	10.95	219
1974	-20.32	12.61	250	-19.70	11.54	219
1975	-19.79	13.41	249	-20.35	12.31	206
1976	-17.76	12.64	247	-16.75	10.82	210
1977	-17.02	10.35	243	-16.01	10.82	221
1978	-18.79	11.79	239	-20.34	10.34	200
1979	-16.81	11.81	238			212
1980	-15.41	11.72	248	-16.68	10.72	186
1981	-19.47	11.32	231	-17.92	12.21	213
1982	-21.29	12.70	249	-20.44	10.37	208
1983	-20.25	11.23	231	-18.06	10.87	206
1984	-20.28	11.34	236	-19.37	11.02	212
1985	-18.01	11.80	239	-18.72	8.73	206
1986	-17.71	11.59	250	-15.80	10.91	227
1987	-17.71	11.06	222	-17.42	10.30	191
1988	-17.52	12.41	239	-18.70	10.62	202
1989	-20.17	13.25	230	-18.81	13.22	209
1990	-20.34	12.16	219	-19.14	10.96	201
1991	-19.94	10.89	232	-18.34	10.77	219
1992			261			219
1993	-18.35	12.72	223			197
1994	-18.84	11.37	218	-18.84	9.71	189
1995	-18.50	11.17	230	-17.93	11.32	220
1996			241	-18.51	10.27	215
1997	-15.31	11.85	228	-16.46	11.19	199
1998	-17.62	10.33	226	-15.67	11.77	221
1999	-16.91	11.03	258	-16.78	10.03	216
2000	-16.11	10.00	255	-15.64	9.97	225
2001	-17.78	9.83	224	-17.29	10.18	214
2002	-14.67	10.89	243	-15.99	9.64	195
2003	-16.74	12.98	257	-17.89	11.21	207
2004	-17.59	10.26	230	-17.84	9.72	200
2005	-16.08	9.95	239	-16.37	10.58	208
2006	-17.31	9.98	228	-17.70	11.11	207
2007	-16.19	10.85	243	-17.75	10.80	216
2008	-16.81	11.00	241	-18.73	11.21	201
2009	-15.11	10.43	237	-16.04	9.73	196
2010	-15.64	9.44	241	-15.98	11.38	221
2011	-17.67	11.02	230	-16.40	11.09	217
2012	-18.98	10.40	231			219
2013	-16.52	10.23	199	-18.31	11.48	197
2014	-15.06	9.54	222	-17.19	9.71	191
2015	-14.06	9.84	231	-14.72	8.76	206
2016	-15.55	9.15	224	-16.17	9.60	216
2017	-12.62	9.57	238	-15.23	9.98	207
2018	-12.31	8.74	236	-12.78	10.89	221

Table S4. Freezing days and mean temperature for freezing periods. Source environment Canda.

	Discharge m^3/s	DOC mg/L	DOC-$F^{14}C$	NO_3^- mg/L	SO_4^{2-} mg/L	Cl^- mg/L	Na^+ mg/L	K^+ mg/L	Mg^{2+} mg/L	Ca^{2+} mg/L
Discharge	-									
ln(DOC)	**0.59*** **	-								
DOC-$F^{14}C$	0.11^{ns}	-0.06^{ns}	-							
ln(NO_3^-)	**-0.57*** **	**-0.47*** **	-0.06^{ns}	-						
SO_4^{2-}	**-0.57*** **	**-0.56*** **	-0.10^{ns}	0.28*	-					
ln(Cl^-)	**-0.85*** **	**-0.45*** **	0.01^{ns}	**0.49*** **	**0.60*** **	-				
ln(Na^+)	**-0.94*** **	**-0.55*** **	-0.12^{ns}	**0.54*** **	**0.54*** **	**0.87*** **	-			
K^+	**-0.61*** **	-0.08^{ns}	0.21^{ns}	0.24*	0.08^{ns}	**0.67*** **	**0.66*** **	-		
Mg^{2+}	**-0.77*** **	**-0.52*** **	0.09^{ns}	0.36**	**0.63*** **	**0.58*** **	**0.78*** **	0.36**	-	
ln(Ca^{2+})	**-0.69*** **	**-0.46*** **	-0.15^{ns}	0.31*	0.37**	**0.45*** **	**0.73*** **	**0.42*** **	**0.87*** **	-

Table S5. Relationships between water chemistry parameters for 87 samples (66 PARTNERS/ArcticGRO, 21 this study) of the Mackenzie River at Tsiigehtchic. Correlations are reported as Pearson product-moment correlation coefficient and significant *p*-value ($p < 0.05$, *), ($p < 0.01$, **) or ($p < 0.001$,***) or as not significant (ns). Relationships were visually inspected and transformed when necessary to meet the assumption of linearity (natural logarithmic transformation are noted in the column on the left).

Total burned area						
Year	total Mackenzie Basin		Peel Basin		Arctic Red Basin	
	km^2	%	km^2	%	km^2	%
2015	23307.39	1.29	49.89	0.06	0	0
2016	11198.65	0.62	0	0	0	0
2017	12131.50	0.67	2141.31	2.65	99.57	0.46
2018	5500.68	0.30	2.37	0	9.19	0.04

Table S6. Total burned area in km^2 and the percentage of burned area for the Mackenzie, the Peel and the Arctic Red River basins.
