# Supplementary material for: An Abrupt Aging of Dissolved Organic Carbon in Large Arctic Rivers
Source: Geophys Res Lett. 2020 Dec 8;47(23):e2020GL088823. doi: 10.1029/2020GL088823 (PMC7757186; doi:10.1029/2020GL088823)
Supplement: Supplementary file 2 — Table S1 [file GRL-47-e2020GL088823-s002.pdf]

Sample ID	River	Location	Date	Latitude	Longitude	Depth (m)	Discharge (m^3/s)	Water yield (mm/d)	DOC (mg/L)			ETH-Nr.	DOC-$F^{14}C$			POC (mg/L)	ETH-Nr.	POC-$F^{14}C$		
CAN17-01	Mackenzie	Delta	03 June 2017	68.4177	134.1149	6	18368	0.92	8.35	±	0.20	88156.1.1	1.01	±	0.01	4.80	89421.1.1	0.289	±	0.004
CAN17-02	Mackenzie	Delta	03 June 2017	68.4177	134.1160	12	"	"	8.67	±	0.16	88157.1.1	1.02	±	0.01	6.52	89422.1.1	0.300	±	0.004
CAN17-03	Mackenzie	Delta	03 June 2017	68.4178	134.1144	18	"	"	8.64	±	0.13	88158.1.1	0.99	±	0.01	7.19	89423.1.1	0.321	±	0.005
CAN17-04	Mackenzie	Delta	03 June 2017	68.4178	134.1160	0	"	"	8.84	±	0.25	88159.1.1	0.96	±	0.01	4.03	89424.1.1	0.283	±	0.004
CAN17-05-08	Mackenzie	Delta	03 June 2017	68.4137	134.1153	10	"	"	7.76	±	0.04	88160.1.1	1.02	±	0.01	32.06	89425.1.1	0.597	±	0.006
CAN17-09	Mackenzie	Delta	03 June 2017	68.4137	134.1152	4	"	"								6.02	89426.1.1	0.320	±	0.002
CAN17-10	Mackenzie	Delta	03 June 2017	68.4135	134.1162	0	"	"	8.42	±	0.10	88161.1.1	1.03	±	0.01	4.11	89427.1.1	0.289	±	0.004
CAN17-12	Mackenzie	Tsiigehtchic	05 June 2017	67.4540	133.7371	19	15746	0.81	7.39	±	0.07	88162.1.1	1.02	±	0.01	4.22	89428.1.1	0.365	±	0.003
CAN17-13	Mackenzie	Tsiigehtchic	05 June 2017	67.4536	133.7368	15	"	"								3.18	89429.1.1	0.364	±	0.003
CAN17-14	Mackenzie	Tsiigehtchic	05 June 2017	67.4537	133.7366	10	"	"	7.59	±	0.09	88163.1.1	1.02	±	0.01	4.10	89430.1.1	0.360	±	0.005
CAN17-15	Mackenzie	Tsiigehtchic	05 June 2017	67.4535	133.7368	5	"	"								3.42	89431.1.1	0.363	±	0.005
CAN17-16	Mackenzie	Tsiigehtchic	05 June 2017	67.4531	133.7366	0	"	"	7.74	±	0.14	93043.1.1	1.03	±	0.02	2.63	89432.1.1	0.361	±	0.003
CAN17-17-20	Mackenzie	Tsiigehtchic	05 June 2017	67.4531	133.7270	9	"	"	8.13	±	0.57	88165.1.1	0.99	±	0.01	12.89	89433.1.1	0.461	±	0.004
CAN17-21	Mackenzie	Tsiigehtchic	05 June 2017	67.4528	133.7270	4	"	"	7.34	±	0.12	88166.1.1	1.01	±	0.01	6.18	89434.1.1	0.353	±	0.005
CAN17-22	Mackenzie	Tsiigehtchic	05 June 2017	67.4528	133.7270	0	"	"	7.93	±	0.15	88167.1.1	1.01	±	0.01	3.87	89435.1.1	0.395	±	0.005
CAN17-26-29	Arctic Red	Tsiigehtchic	05 June 2017	67.4298	133.7784	4	512.65	1.91	11.35	±	0.56	88168.1.1	1.00	±	0.01	15.33	89436.1.1	0.451	±	0.006
CAN17-30	Arctic Red	Tsiigehtchic	05 June 2017	67.4299	133.7788	0	"	"	11.42	±	0.18	88169.1.1	0.98	±	0.01	8.76	89437.1.1	0.302	±	0.004
CAN17-33-36	Peel	Fort McPherson	07 June 2017	67.3289	134.8662	7	1303	1.59	3.64	±	0.05	88170.1.1	0.98	±	0.02	35.52	89438.1.1	0.611	±	0.007
CAN17-37	Peel	Fort McPherson	07 June 2017	67.3288	134.8665	4	"	"	4.30	±	0.06	88171.1.1	0.98	±	0.02	8.16	89439.1.1	0.470	±	0.004
CAN17-38	Peel	Fort McPherson	07 June 2017	67.3287	134.8669	2	"	"	3.98	±	0.27					7.20	89440.1.1	0.435	±	0.006
CAN17-39	Peel	Fort McPherson	07 June 2017	67.3282	134.8664	0	"	"	3.94	±	0.06	88173.1.1	0.93	±	0.02	3.74	89441.1.1	0.338	±	0.005
CAN17-46-49	Mackenzie	Delta	10 June 2017	68.4151	134.1209	20	16647	0.84	7.21	±	0.06	88174.1.1	0.98	±	0.01	29.91	89442.1.1	0.589	±	0.006
CAN17-50	Mackenzie	Delta	10 June 2017	68.4153	134.1179	18	"	"	7.01	±	0.09	88175.1.1	1.00	±	0.01	6.97	89443.1.1	0.380	±	0.004
CAN17-51	Mackenzie	Delta	10 June 2017	68.4154	134.1184	12	"	"	6.57	±	0.58	88176.1.1	1.02	±	0.02	5.73	89444.1.1	0.367	±	0.004
CAN17-52	Mackenzie	Delta	10 June 2017	68.4154	134.1173	6	"	"	6.55	±	0.05	93045.1.1	1.06	±	0.02					
CAN17-53	Mackenzie	Delta	10 June 2017	68.4154	134.1173	0	"	"	6.21	±	0.10	88178.1.1	0.98	±	0.01	1.82	89446.1.1	0.324	±	0.004
CAN18-02	Mackenzie	Delta	06 June 2018	68.4178	134.1116	0	14670	0.74	9.44	±	0.06	93133.1.1	0.61	±	0.01	1.41	92585.1.1	0.361	±	0.006
CAN18-02 rep	Mackenzie	Delta	06 June 2018	68.4178	134.1116	0	"	"				98948.1.1	0.60	±	0.01					
CAN18-03	Mackenzie	Delta	06 June 2018	68.4137	134.1164	10	"	"	7.42	±	0.13	93134.1.1	0.79	±	0.01	6.61	92586.1.1	0.528	±	0.005
CAN18-04	Mackenzie	Delta	06 June 2018	68.4141	134.1155	14	"	"								6.53	92587.1.1	0.522	±	0.005
CAN18-05-11	Mackenzie	Delta	06 June 2018	68.4138	134.1134	11	"	"								9.95	92588.1.1	0.568	±	0.006
CAN18-12	Mackenzie	Delta	06 June 2018	68.4137	134.1117	7	"	"	6.23	±	0.05	93135.1.1	0.98	±	0.02	4.28	92589.1.1	0.447	±	0.005
CAN18-12 rep	Mackenzie	Delta	06 June 2018	68.4137	134.1117	7	"	"				98949.1.1	0.98	±	0.02					
CAN18-14	Mackenzie	Delta	08 June 2018	68.4171	134.1150	0	"	"	6.60	±	0.04	93136.1.1	0.93	±	0.02		92590.1.1	0.366	±	0.004
CAN18-18	Mackenzie	Tsiigehtchic	09 June 2018	67.4530	133.7254	8	14010	0.72								6.91	92591.1.1	0.536	±	0.006
CAN18-19-25	Mackenzie	Tsiigehtchic	09 June 2018	67.4535	133.7233	8	"	"	7.10	±	0.02	93046.1.1	0.73	±	0.01	10.28	92592.1.1	0.570	±	0.006
CAN18-26	Mackenzie	Tsiigehtchic	09 June 2018	67.4564	133.7240	14	"	"	6.40	±	0.12	93047.1.1	0.87	±	0.02					
CAN18-28	Mackenzie	Tsiigehtchic	09 June 2018	67.4567	133.7223	10	"	"	5.56	±	0.09	93137.1.1	1.02	±	0.03	2.29	92594.1.1	0.453	±	0.005
CAN18-29	Mackenzie	Tsiigehtchic	09 June 2018	67.4567	133.7223	5	"	"	5.10	±	0.06	93138.1.1	0.97	±	0.03	1.66	92595.1.1	0.448	±	0.005
CAN18-31	Mackenzie	Tsiigehtchic	09 June 2018	67.4562	133.7234	0	"	"	5.46	±	0.08	93139.1.1	0.99	±	0.02	1.52	92596.1.1	0.433	±	0.005
CAN18-31 rep	Mackenzie	Tsiigehtchic	09 June 2018	67.4562	133.7234	0	"	"				98950.1.1	0.98	±	0.02					
CAN18-32	Arctic Red	Tsiigehtchic	09 June 2018	67.4263	133.7789	9	369	1.37	8.24	±	0.03	93048.1.1	0.51	±	0.01	7.62	92597.1.1	0.351	±	0.004
CAN18-32 rep	Arctic Red	Tsiigehtchic	09 June 2018	67.4263	133.7789	9	"	"				98951.1.1	0.51	±	0.01					
CAN18-36	Arctic Red	Tsiigehtchic	09 June 2018	mix	mix	9	"	"								7.49	92598.1.1	0.351	±	0.004
CAN18-37	Arctic Red	Tsiigehtchic	09 June 2018	67.4261	133.7786	4	"	"	6.67	±	0.08	93049.1.1	0.67	±	0.01	6.52	92599.1.1	0.308	±	0.003
CAN18-38	Arctic Red	Tsiigehtchic	09 June 2018	67.4265	133.7790	0	"	"	5.86	±	0.15	93140.1.1	0.97	±	0.03	4.19	92600.1.1	0.257	±	0.003
CAN18-49	Peel	Fort McPherson	12 June 2018	67.3245	134.8635	7	1614	1.97								30.06	92601.1.1	0.451	±	0.005
CAN18-51	Peel	Fort McPherson	12 June 2018	mix	mix	7	"	"	3.25	±	0.04	93051.1.1	0.85	±	0.03	24.36	92602.1.1	0.409	±	0.004
CAN18-52	Peel	Fort McPherson	12 June 2018	67.3252	134.8635	6	"	"	3.16	±	0.06	93141.1.1	0.89	±	0.04	5.58	92603.1.1	0.390	±	0.004
CAN18-53	Peel	Fort McPherson	12 June 2018	67.3253	134.8642	2	"	"	3.82	±	0.04	93142.1.1	0.68	±	0.03	4.09	92604.1.1	0.298	±	0.003
CAN18-54	Peel	Fort McPherson	12 June 2018	67.3260	134.8640	0	"	"	3.58	±	0.06	93052.1.1	0.67	±	0.02	3.01	92605.1.1	0.275	±	0.003
CAN18-55	Arctic Red	Tsiigehtchic	08 May 2018	67.4444	133.7473	0			2.91	±	0.04	93053.1.1	0.95	±	0.04					
CAN18-56	Arctic Red	Tsiigehtchic	11 May 2018	67.4444	133.7473	0			11.71	±	0.10	93143.1.1	0.99	±	0.02	4.98	92606.1.1	0.389	±	0.004
CAN18-57	Arctic Red	Tsiigehtchic	14 May 2018	67.4444	133.7473	0			11.58	±	0.10	93054.1.1	0.85	±	0.01	2.78	92607.1.1	0.340	±	0.003
CAN18-58	Arctic Red	Tsiigehtchic	17 May 2018	67.4444	133.7473	0			9.64	±	0.09	93055.1.1	1.06	±	0.02	2.64	92608.1.1	0.513	±	0.005
CAN18-59	Arctic Red	Tsiigehtchic	20 May 2018	67.4444	133.7473	0			11.97	±	0.22	93144.1.1	0.93	±	0.01	7.86	92609.1.1	0.371	±	0.004
CAN18-60	Arctic Red	Tsiigehtchic	23 May 2018	67.4444	133.7473	0			11.28	±	0.13	93056.1.1	0.94	±	0.01	8.45	92610.1.1	0.358	±	0.003
CAN18-61	Arctic Red	Tsiigehtchic	26 May 2018	67.4444	133.7473	0			7.81	±	0.13	93145.1.1	1.03	±	0.02	4.93	92611.1.1	0.329	±	0.004
CAN18-62	Arctic Red	Tsiigehtchic	29 May 2018	67.4444	133.7473	0			9.94	±	0.16	93146.1.1	0.89	±	0.01	5.26	92612.1.1	0.282	±	0.003
CAN19-03	Mackenzie	Delta	04 June 2019	68.4179	134.1158	0	13358	0.67	6.75	±	0.03	98926.1.1	1.04	±	0.02	1.66	101975.1.1	0.396	±	0.004
CAN19-04	Mackenzie	Delta	04 June 2019	68.4172	134.1163	9	"	"	6.04	±	0.07	98927.1.1	1.04	±	0.02	2.46	101976.1.1	0.394	±	0.004
CAN19-05	Mackenzie	Delta	04 June 2019	68.4172	134.1195	17	"	"	6.66	±	0.04	98928.1.1	1.06	±	0.02	3.35	101977.1.1	0.391	±	0.004
CAN19-06	Mackenzie	Delta	04 June 2019	68.4176	134.1275	29	"	"	6.88	±	0.02	98929.1.1	1.04	±	0.02	4.31	101978.1.1	0.383	±	0.004
CAN19-07	Mackenzie	Delta	04 June 2019	68.4179	134.1159	0	"	"	7.20	±	0.11	98930.1.1	1.02	±	0.02	1.53	101979.1.1	0.405	±	0.004
CAN19-08	Mackenzie	Delta	04 June 2019	68.4179	134.1173	15	"	"	6.09	±	0.06	98931.1.1	1.05	±	0.02	2.88	101980.1.1	0.424	±	0.004
CAN19-09	Mackenzie	Delta	04 June 2019	68.4177	134.1211	28	"	"	6.57	±	0.12	98932.1.1	1.06	±	0.02	3.08	101981.1.1	0.387	±	0.004
CAN19-14	Peel	Fort McPherson	08 June 2019	67.3384	134.8770	0			5.12	±	0.06	98934.1.1	1.03	±	0.02	2.67	101983.1.1	0.405	±	0.004
CAN19-19	Mackenzie	Tsiigehtchic	10 June 2019	67.4556	133.7344	0	13823	0.71	6.05	±	0.12	98937.1.1	1.05	±	0.03	2.80	101986.1.1	0.407	±	0.004
CAN19-20	Mackenzie	Tsiigehtchic	10 June 2019	67.4555	133.7350	6	"	"	5.16	±	0.12	98938.1.1	1.04	±	0.02	3.81	101987.1.1	0.419	±	0.004
CAN19-21	Mackenzie	Tsiigehtchic	10 June 2019	67.4553	133.7356	14	"	"	5.64	±	0.11	98939.1.1	1.04	±	0.02	3.36	101988.1.1	0.414	±	0.004
CAN19-22	Mackenzie	Tsiigehtchic	10 June 2019	67.4551	133.7368	19	"	"	5.91	±	0.04					3.97	101989.1.1	0.393	±	0.004
CAN19-23	Mackenzie	Tsiigehtchic	10 June 2019	67.4539	133.7304	0	"	"	5.96	±	0.07	98941.1.1	1.05	±	0.02	3.00	101990.1.1	0.412	±	0.004
CAN19-24	Mackenzie	Tsiigehtchic	10 June 2019	67.4537	133.7316	6	"	"	5.45	±	0.06	98942.1.1	1.05	±	0.02	4.15	101991.1.1	0.430	±	0.004
CAN19-25	Mackenzie	Tsiigehtchic	10 June 2019	67.4537	133.7316	11	"	"	6.02	±	0.11	98943.1.1	1.04	±	0.02	7.91	101992.1.1	0.640	±	0.007
CAN19-32	Arctic Red	Tsiigehtchic	10 June 2019	67.4263	133.7790	0	235	0.88	11.17	±	0.15	98944.1.1	1.01	±	0.01	2.46	101993.1.1	0.459	±	0.005
CAN19-33	Arctic Red	Tsiigehtchic	10 June 2019	67.4263	133.7790	2	"	"	10.40	±	0.13	98945.1.1	0.99	±	0.01	2.67	101994.1.1	0.458	±	0.004
CAN19-34	Arctic Red	Tsiigehtchic	11 June 2019	67.4265	133.7798	4	"	"	10.86	±	0.21	98946.1.1	1.01	±	0.01	2.72	101995.1.1	0.481	±	0.005

Table S1. River suspended dissolved and particulate material.
